# Induced B cell receptor diversity predicts PD-1 blockade immunotherapy response

**DOI:** 10.1073/pnas.2501269122

**Published:** 2025-05-02

**Authors:** Yonglu Che, Jinwoo Lee, Farah Abou-Taleb, Kerri E. Rieger, Ansuman T. Satpathy, Anne Lynn S. Chang, Howard Y. Chang

**Affiliations:** ^a^Department of Dermatology, Stanford University School of Medicine, Redwood City, CA 94063; ^b^Department of Pathology, Stanford University School of Medicine, Stanford, CA 94304; ^c^Department of Genetics, Stanford University School of Medicine, Stanford, CA 94305; ^d^HHMI, Stanford University School of Medicine, Stanford, CA 94305

**Keywords:** immunotherapy, spatial transcriptomics, cancer, basal cell carcinoma, PD-1 inhibition

## Abstract

Immune checkpoint inhibitors such as anti-Programmed Death-1 antibodies (aPD-1) can be effective in treating advanced cancers. However, many patients do not respond, and the mechanisms underlying these differences remain incompletely understood. In this study, we profile a cohort of patients with locally advanced or metastatic basal cell carcinoma undergoing aPD-1 therapy using single-cell RNA sequencing, high-definition spatial transcriptomics in tumors and draining lymph nodes, and spatial immunoreceptor profiling, with long-term clinical follow-up. We find that successful responses to PD-1 inhibition are characterized by an induction of B cell receptor (BCR) clonal diversity after treatment initiation. These induced BCR clones spatially colocalize with T cell clones, facilitate their activation, and traffic alongside them between tumor and draining lymph nodes to enhance tumor clearance. Furthermore, we validated aPD-1-induced BCR diversity as a predictor of clinical response in a larger cohort of glioblastoma, melanoma, and head and neck squamous cell carcinoma patients, suggesting that this is a generalizable predictor of treatment response across many types of cancers. We find that pretreatment tumors harbor a characteristic gene expression signature that portends a higher probability of inducing BCR clonal diversity after aPD-1 therapy, and we develop a machine learning model that predicts PD-1-induced BCR clonal diversity from baseline tumor RNA sequencing. These findings underscore a dynamic role of B cell diversity during immunotherapy, highlighting its importance as a prognostic marker and a potential target for intervention in non-responders.

## Significance

Checkpoint blockade has transformed cancer immunotherapy, but many patients do not respond clinically. In this work, we longitudinally profile a cohort of patients with locally advanced skin cancers before, during, and after checkpoint blockade. By tracking single-cell and spatial immunoreceptor sequences and transcriptome in both multiple tumor biopsies and the draining lymph nodes, we found that anti-Programmed Death (aPD)-1 induced B cell clonal diversity is critical for T cell expansion, activation, and significantly improved patient overall survival. This dataset is one of the most comprehensive and informative datasets for human cancer immunotherapy, as paired tumor biopsies and on-therapy lymph node biopsies are extremely important but rare.

Immune checkpoint inhibitors, including programmed cell death protein 1 (PD-1) inhibitors, have significantly improved long-term outcomes of patients with advanced cancer. Despite this success, patient responses are highly variable, and the underlying mechanisms of a successful response are incompletely understood (1–3). Many studies have attempted to identify predictors of treatment response, including tumor expression of PD-L1, PD-L2, IFNγ, CD73, and CXCL9, as well as tumor mutation burden and the formation of tertiary lymphoid structures (TLSs), but these do not fully explain the variability of patient responses (4–6).

The tumor microenvironment is highly dynamic. Contrary to the classical view of immunologically “hot” vs. “cold” tumors defined by the mere presence or absence of T cell infiltration, we and others found that anti-PD-1 (aPD-1) checkpoint blockade induced clonal replacement of T cells, subsequent expansion detectable in the peripheral blood, migration into the tumor, followed by multiple rounds of activation, expansion, and ultimately T cell exhaustion (7, 8). These dynamic changes highlight the importance of immune cell trafficking and nominate tumor-draining lymph nodes as compelling sites of cell–cell interactions (9). Recent evidence suggests that B cells play a crucial role in the anti-PD-1 response. In melanoma patients, B cell markers are enriched in responders and localize to tumor TLSs (10). Similarly, in soft tissue sarcomas, B cell–rich TLSs were associated with better prognosis (11, 12). Additional studies have implicated specific B cell subtypes as positive prognostic indicators in multiple cancer types (13–20), further highlighting the importance of B cells in antitumor immunity. Questions remain, however, regarding the mechanisms behind these findings and how these insights might translate into clinical practice.

Building on our previous longitudinal analysis, which examined the role of novel tumor-infiltrating T cell clonotypes in reinvigorating antitumor responses following PD-1 blockade in advanced cutaneous basal cell carcinoma (BCC) and cutaneous squamous cell carcinoma (SCC) (7), we performed long-term clinical outcome monitoring, single cell transcriptome and immunoreceptor sequencing, and high-definition spatial transcriptomics of tumors and draining lymph node specimens collected over years of direct patient care. Because T-cell receptor and B-cell receptor (BCR) genes undergo somatic recombination, TCR and BCR sequences provide unique molecular barcodes that are passed onto their progenies, allowing us to track the clonal dynamics during checkpoint blockade in patients. By integrating single-cell and spatial transcriptomic profiling with clinical outcomes, we reveal a significant role for increased BCR clonal diversity following PD-1 blockade. Our findings demonstrate that diverse B cell clones migrate and interact with specific T cells in both the tumoral microenvironment and within the draining lymph nodes to enhance T cell expansion and activation, ultimately improving tumor clearance.

## Results

### Patient Survival after PD-1 Inhibition in BCC Is Associated with Intratumoral BCR Diversification.

**Fig. 1.**
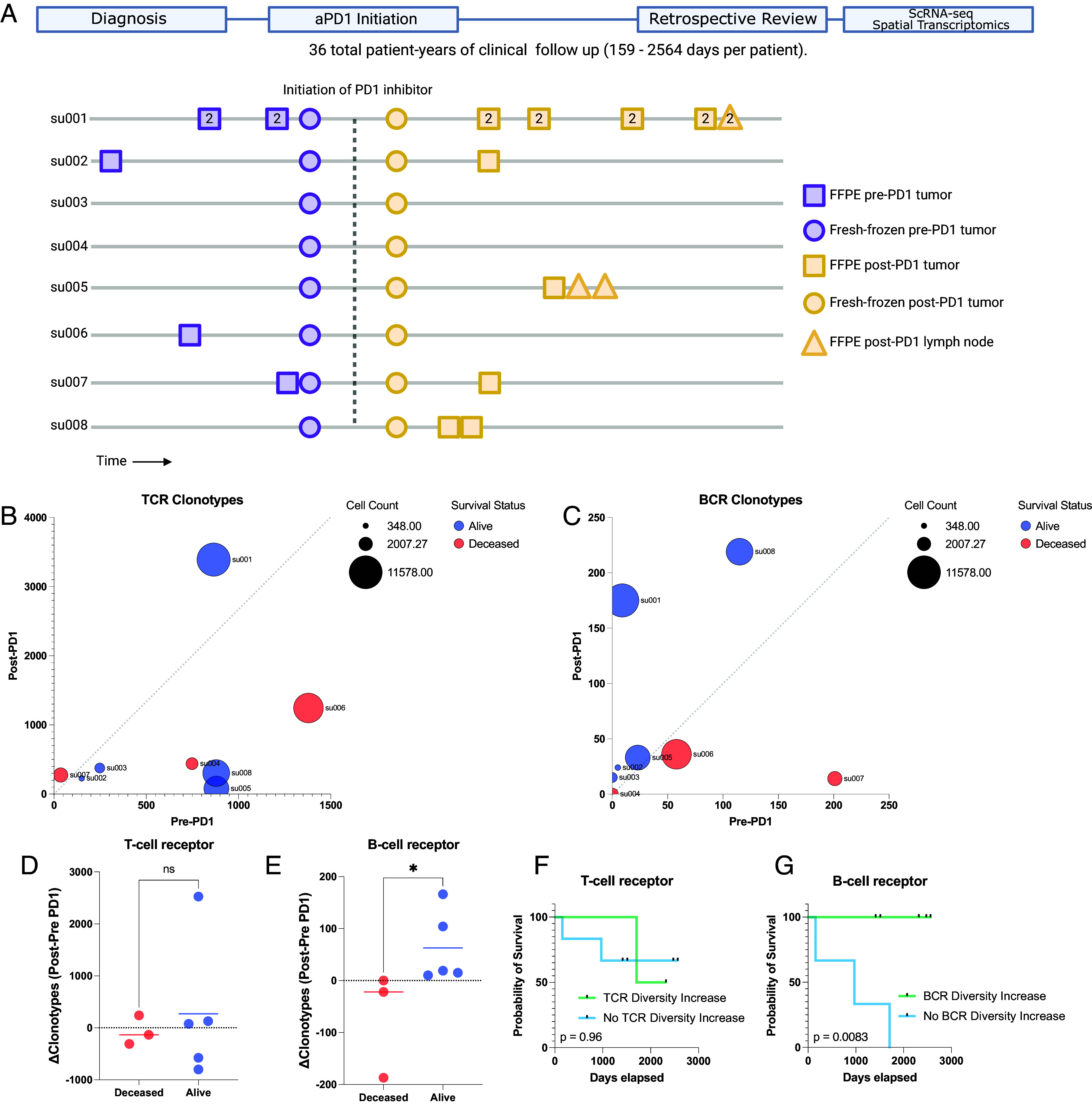
Long-term survival of advanced BCC patients highlights prognostic role of aPD-1- induced BCR clonal diversity. (*A*) A schematic diagram of our BCC patient cohort. (*Top*) A retrospective chart review was performed after an extended period of clinical follow-up lasting up to 2,564 d to allow final clinical outcomes of PD-1 blockade to manifest. (*Bottom*) A visualization of the samples taken for our prior study (7) and this study. All patients had pre- and post-PD-1 inhibitor-treated tumor samples for scRNA-seq (circles). All available archived specimens in long-term storage were accessed (squares and triangles) and processed for spatial transcriptomics. Numbers within the shapes represent replicates taken from sequential sections of the sample tissue block. The *y*-axis represents anonymized patient identifiers while the x-axis represents time (not to exact scale). (*B*) Unique TCR and (*C*) BCR clonotype counts detected per patient in their pre-PD-1 inhibitor tumor (*X*-axis) and post-PD-1 inhibitor treated tumor (*Y* axis) as calculated by TRUST4 run on 5′ single cell RNA sequencing as described (*Materials and Methods*). Point sizes represent the total cell count obtained from tumors of each patient. (*D*) Change in unique TCR and (*E*) BCR clonotype counts between pre-PD-1 inhibitor and post-PD-1 inhibitor tumors for each patient (*Y*-axis), stratified by clinical status at last available follow-up. (*F*) Kaplan–Meier curve of overall survival for our BCC patient cohort stratified by the change in pre-to-post PD-1 inhibitor TCR and (*G*) BCR clonotype count. *P*-values calculated using the log-rank test.

Previous work from our group led to the discovery that expansion of tumor-infiltrating T lymphocytes arises from novel rather than preexisting clones in a cohort of locally advanced and metastatic cutaneous carcinoma patients treated with PD-1 inhibitors (7). To gain further insight into the mechanisms that influence long-term survival after treatment, we extended the clinical follow-up of this patient cohort and archived additional samples taken for clinical care during this time for future analysis (Fig. 1*A* and Dataset S1). To investigate B cell clonality within our cohort, we analyzed single-cell RNA sequencing data to identify BCR sequences and quantify the number of unique clonotypes per tumor specimen (Fig. 1 *B* and *C* and *SI Appendix*, Fig. S1 *A*–*F*). The sequencing datasets were prepared using 5′ gene expression library construction as previously described and sequenced to a minimum depth of 25,000 reads per cell. Using TRUST4 for clonotype calling analogous to previous work in this field (10, 21), we find that the complementarity-determining region 3 (CDR3) sequence lengths in our analysis displayed comparable distributions to known BCR and TCR lengths (22).

Analysis of the TCR and BCR repertoires revealed considerable heterogeneity both among patients and between pre- and post-PD-1 inhibitor treatment tumor specimens from the same patient (Fig. 1 *B* and *C*), suggesting a level of variability in the tumor-infiltrating lymphocyte population. For clarity, we refer to “clonal expansion” or “clone size” as the number of T or B cells with the same immunoreceptor sequences, while “clonal diversity” refers to the number of T or B cells with distinct immunoreceptor sequences. TCR clonal expansion and TCR diversity were not directly associated with improved survival in this cohort (Fig. 1*D* and *SI Appendix*, Fig. S1*G*). In contrast, while BCR clonotype counts at individual time points did not correlate with survival outcomes, we observed an increase in unique BCR clonotype counts after PD-1 blockade exclusively in surviving patients (Fig. 1*E*). Patients with an increase in intratumoral BCR clonotype diversity demonstrated a markedly improved survival rate compared to those without expansion (log-rank test *P* = 0.0083, Fig. 1 *F* and *G* and *SI Appendix*, Fig. S1*H*), which contrasts with the lack of clear association between either TCR clonal expansion or TCR clonal diversity with survival outcomes. This led us to further investigate the mechanisms by which *PD-1 inhibition induces BCR diversity*.

### Targeted Spatial Transcriptomics of Archived Patient Specimens before and after PD-1 Blockade Captures Evolution of the Tumor and Draining Lymph Node Microenvironment.

To further explore mechanisms by which BCR clonotype diversity might enhance patient survival, we expanded our molecular profiling to include spatial transcriptomics on available specimens. Prior studies have highlighted the utility of spatial data to elucidate T/B cell antitumor mechanisms (17), so we reasoned that this type of dataset would complement our existing scRNA-seq data. We selected the Xenium in situ platform due to its 1) compatibility with formalin-fixed, paraffin-embedded (FFPE) archival specimens, 2) accurate cell segmentation, and 3) flexible custom probe design, which were critical features for our study (23). Recognizing the platform’s current limitations in gene coverage, we carefully designed a custom probe panel to minimize information loss associated with this constraint (*Materials and Methods*). This was accomplished by constructing a 480 gene custom panel comprising the most differentially expressed genes in our scRNA sequencing, essential markers of cell type and cell state, and probes targeting TCR and BCR loci (detailed further below, *Materials and Methods*, *SI Appendix*, Fig. S2*A* and Dataset S2). To objectively evaluate this manually curated design, we then separately trained a neural net model to evaluate the panel’s potential performance. We assigned the model to predict the annotated cell states of the scRNA sequencing data and then measured the change in model accuracy when constrained to limited gene panels including our custom design (*SI Appendix*, Fig. S2*B*). Notably, the 480 gene custom panel showed (under ideal, simulated circumstances) minimal information loss even when compared to the baseline model with full gene expression access and outperformed predesigned panel designs for our application including the recently released Xenium 5K gene panel.

**Fig. 2.**
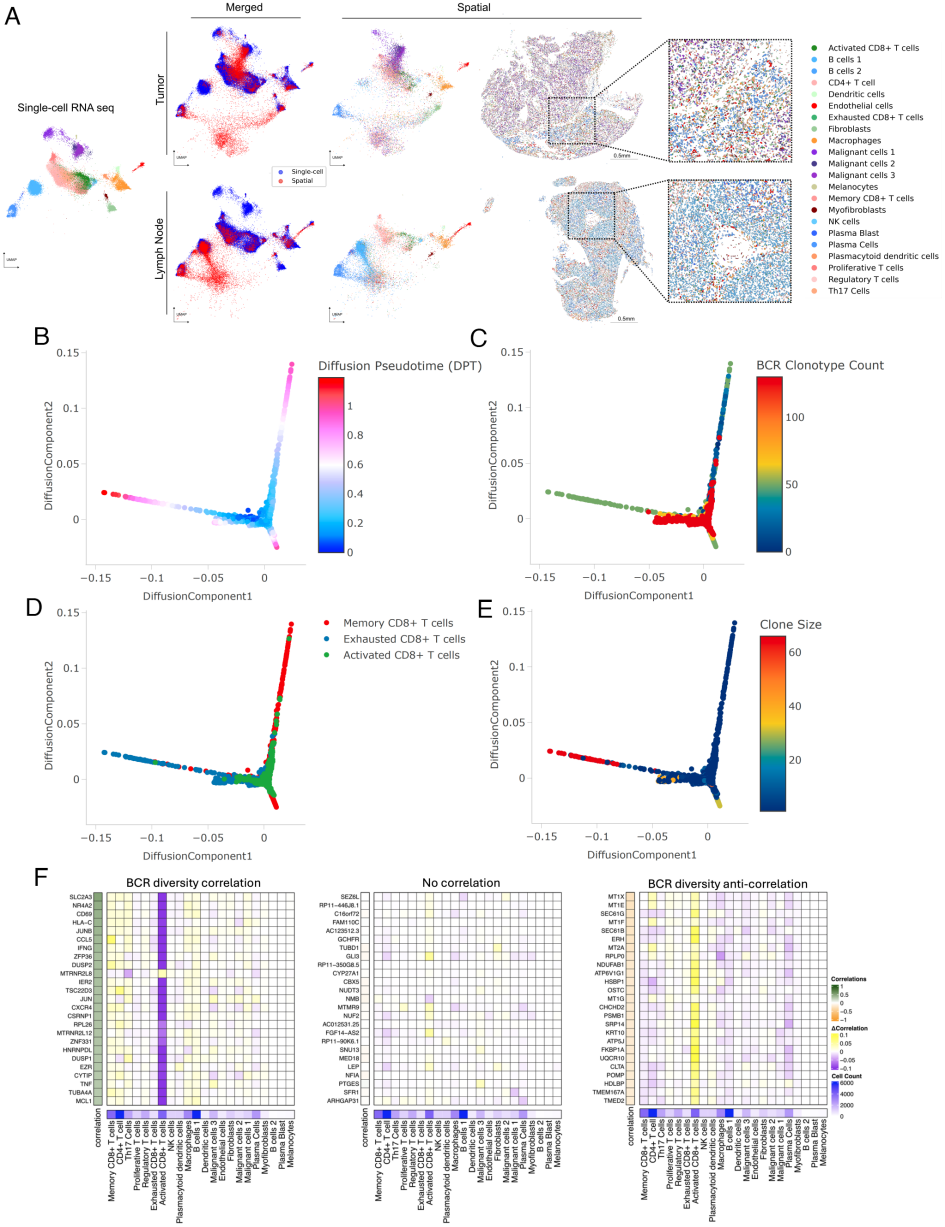
Spatial transcriptomics merged with scRNA-seq enables exploration of the BCR diversity-impacted tumor microenvironment. (*A*) Representative tissue samples obtained from Xenium in situ for patient su001 for tumor (*Top*) and regional lymph node (*Bottom*). Cells from Xenium were merged onto the same latent space (*Middle*) as cells from our prior scRNA-seq (*Left* and *Middle*) using the computational tool ENVI (*Materials and Methods*). Proposed cell types for Xenium cells were determined using a nearest-neighbor approach in the high-dimensional ENVI latent space. Xenium tissues are downsampled to 40% of total cells for visualization purposes. (*B*) Diffusion plots of CD8+ T cell populations from scRNA-seq highlighting pseudotime, (*C*) total unique BCR clonotype count present in the originating tumor of each T cell, (*D*) annotated subtype of each T cell (*Bottom*), (*E*) and size of the originating T cell clonotype. (*F*) Regression analysis of cell-type contributions to gene expression correlations to BCR-clonotype counts. Left heatmap represents the correlation across scRNA-seq tissue specimens between the annotated gene and the unique BCR clonotype count in that tissue specimen. The 25 most correlated genes (*Left*), 25 most anticorrelated genes (*Right*), and 25 random genes (*Middle*) are shown. The middle heatmap grid represents the change in correlation strength of each gene (row) when a particular cluster (column) is blinded to the correlation calculation. The heatmap on the *Bottom* row represents the total cell counts assigned to each cluster to verify that effect sizes are not solely influenced by cluster size.

Our final spatial transcriptomics dataset encompassed 35 individual tissue specimens including 29 tumor specimens (21 samples from 6 aPD-1-treated patients, 8 samples from 4 aPD-1-naive patients), two specimens from a tumor-adjacent immune-related adverse event (from a single patient), and four draining lymph node specimens (from two aPD-1-treated patients), amounting to a total of 1,633,794 spatially resolved individual cells (*SI Appendix*, Fig. S3). Combined with the scRNA sequencing data of 53,030 cells from the same patient cohort, this dataset serves as a high-depth resource for investigating tumor and lymph node evolution during checkpoint blockade. To demonstrate a method for integrated analysis of these two datasets, we employed ENVI—a computational tool designed for multimodal data integration of scRNA sequencing and spatial transcriptomics—to create a shared latent space between spatial and single-cell data (24). This approach demonstrates that cells from both modalities can be merged for simultaneous analysis (Fig. 2*A* and *SI Appendix*, Fig. S4 *A*–*E*), facilitating a more detailed understanding of the tumor microenvironment and lymphatics.

### PD-1-Induced B Cell Diversity Supports Activation of CD8+ T Cells.

To investigate mechanisms by which B cells might influence tumor behavior, we examined relationships between BCR clonotype diversity and tumor composition on a sample-by-sample basis using our scRNA-seq data. This allowed us to explore what correlated shifts in tumor microenvironment might be associated with different degrees of intratumoral BCR clonotype diversity. We isolated keratinocyte-containing cell clusters—which include the malignant BCC cells—and assessed their differentiation trajectories using diffusion maps (25). BCC cells, characterized by stem-like properties resembling progenitor keratinocytes and expressing markers such as epithelial cell adhesion molecule EPCAM/BerEP4, mapped to earlier stages of differentiation in the diffusion plot (*SI Appendix*, Fig. S5*A*). In contrast, terminally differentiating keratinocytes, likely from adjacent normal tissue captured in the samples, mapped to regions expressing higher levels of differentiation marker genes like keratin 1 and involucrin. Keratinocytes from tissue samples with higher BCR clonotype diversity were skewed toward terminal differentiation compared to those from low BCR clonotype diversity samples, suggesting lower tumor cell presence in these samples despite being biopsied from the center of clinically active disease.

We performed a similar analysis on B cells to assess the relationship between clonotype diversity and their differentiation states. Interestingly, samples with high BCR clonotype counts were skewed to B cells (with antigen-presenting phenotypes) rather than plasma cells [with high immunoglobulin (IG) expression] (*SI Appendix*, Fig. S5*B*). These results support the hypothesis that diverse B cell clones are involved in antigen cross-presentation to T cells.

Finally, we examined CD8+ T cells within the diffusion map overlaid with BCR clonotype diversity data. Our previously annotated subcategories of CD8+ T cell clusters—including activated, exhausted, and memory phenotypes—mapped to distinct differentiation trajectories. When overlaid with BCR clonotype counts of the originating samples, this analysis showed strong enrichment of the activated CD8+ T cell phenotype in tumor samples with high BCR clonotype counts, suggesting that B cell clonal diversity and CD8+ T cell activation are closely linked (Fig. 2 *B*–*D* and *SI Appendix*, Fig. S5*C*). When overlaid with T cell clonotype sizes, we find that the clones with the largest sizes belonged to the exhausted CD8+ T cell phenotype, whereas the clone sizes of activated CD8+ T cells tended to be smaller (Fig. 2*E*). We pose that these T cells may be in the process of activation, supported by their diverse B cell environment, while exhausted T cells may be a signature more indicative of prior antitumor activity.

Using established network analysis approaches on our gene expression data, we identify pathways by which clonally diverse B cells can communicate with activated CD8+ T cells, including the major histocompatibility complex class I (MHC-I) pathway (*SI Appendix*, Fig. S6 *A*–*D*), as well as validate the activation of the TCR signaling and activator protein 1 pathways in the activated CD8+ T cell population (*SI Appendix*, Fig. S6 *E* and *F*). To examine the specificity of the relationship between B cells and CD8+ T cell activation, we performed a cluster-based regression analysis. We assessed correlations between gene expression in the scRNA sequencing dataset and the BCR clonotype counts of the originating samples (Fig. 2*F*). Our aim was to determine whether gene expression patterns most correlated with intratumoral BCR clonotype diversity were attributable to a single cell type or influenced by cumulative shifts in multiple cell types. This analysis revealed a striking specificity originating from activated CD8+ T cells. The gene expression module correlating to high tumoral BCR diversity prominently featured the hallmark genes of T cell activation such as CD69 (R = 0.42, FDR < 0.001), IFNG (R = 0.37, FDR < 0.001), and TNF (R = 0.29, FDR < 0.001), reinforcing the potential role of activated CD8+ T cells in mediating the effects of increased BCR diversity within the tumor microenvironment. While these data primarily highlight MHC-I-based interactions, we note that this does not exclude the possibility of concurrent MHC-II-based signals.

### Spatial Transcriptomics Enables Clonotype Tracking to Elucidate T Cell:B Cell Interactions.

The strong correlation between BCR clonotype diversity and gene signature of activated CD8+ T cells, along with lower malignant keratinocyte burden in these BCR-diverse tissues, suggests a mechanism where B cell clonal diversification following PD-1 blockade activates T cells leading to tumor clearance. Further exploration of this mechanism required analysis using spatial transcriptomics. This posed a technical challenge since established spatial technologies for archival FFPE specimens, to our knowledge, do not have the ability to simultaneously map lymphocyte clonotype locations. To overcome this, we further customized our Xenium panel to enable tracking of T and B cell clonotype trends in our tissue specimens.

**Fig. 3.**
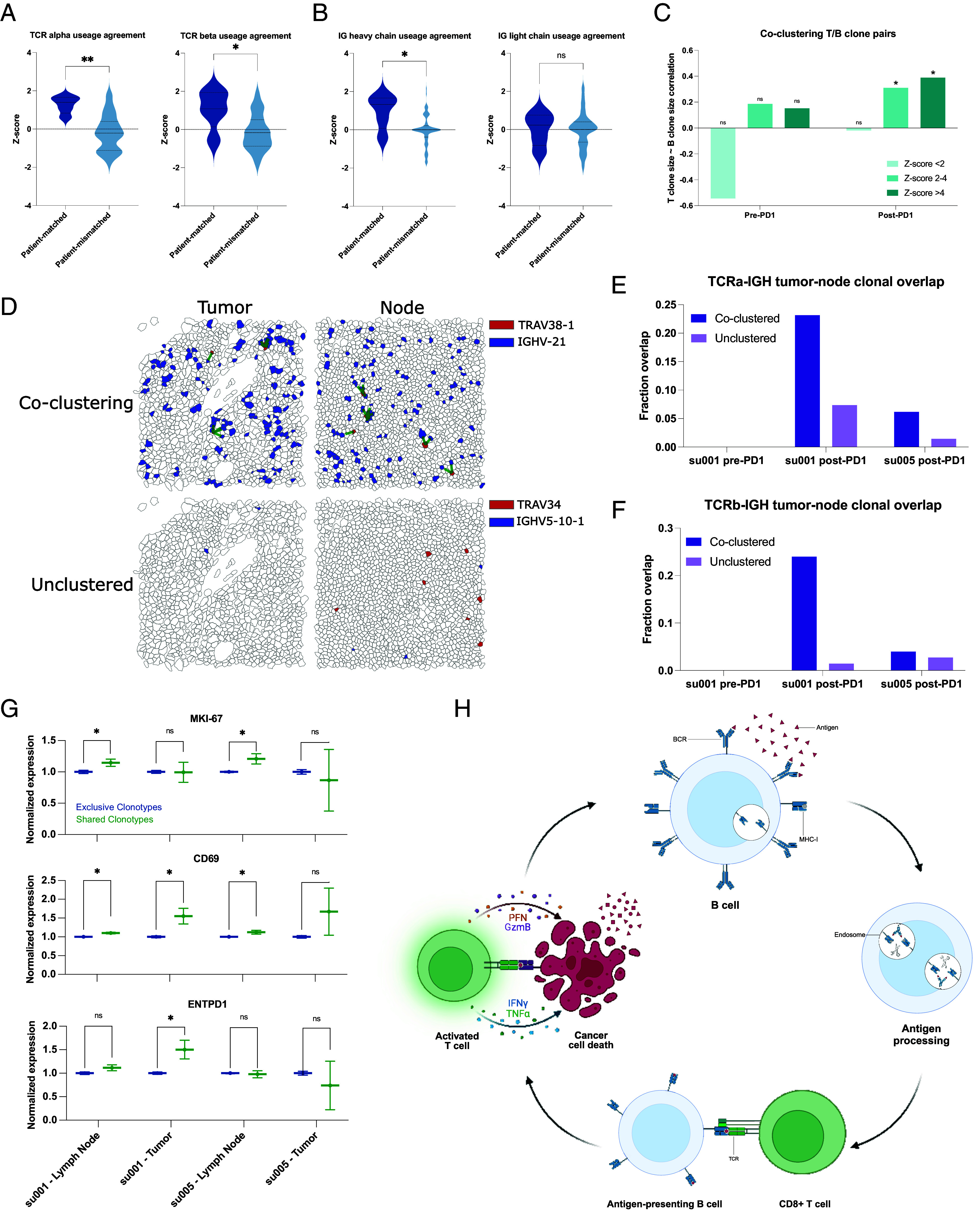
Clonally diverse B cells cocluster with paired T cell clones with an activated phenotype. (*A*) Correlations of V-gene usage between those detected using TRUST4 analysis of scRNA-seq vs. those detected in Xenium in situ for TCRs. “Patient-matched” refers to correlations between Xenium samples and scRNA-seq samples originating from the same patient at the same clinical time point, whereas “patient-mismatched” refers to correlations between different patients or different clinical timepoints. (*B*) Correlations of V-gene usage between scRNA-seq vs. Xenium for IG-genes. (*C*) Correlation of T cell “pseudoclone” sizes with paired B cell pseudoclone sizes as a function of PD-1 inhibitor exposure of the originating tumor tissue and degree of spatial coclustering between those T and B “pseudoclones.” (*D*) Representative images of tumor and lymph node samples from patient su001 highlighting a pair of coclustered pseudoclones and a pair of unclustered pseudoclones. Green lines represent T/B cell clones within 20 µm of each other. (*E*) Fraction of TCRα pseudoclone pairs present in the draining lymph node samples that are also present in the respective tumor sample of that patient. All lymph node samples were collected post-PD-1 inhibitor treatment. Patient su005 had no remaining archived pre-PD-1 inhibitor tumor to be analyzed. (*F*) Fraction of TCRβ pairs present in both draining lymph nodes and tumors. (*G*) Normalized gene expression by patient and tissue compartment for T cell clones that are present in both lymph nodes and tumor (shared) vs. those present in a single compartment (exclusive). (*H*) Schematic diagram of a proposed mechanism of antigen cross-presentation by B cells to CD8+ T cells leading to a cycle of tumor clearance and generation of additional tumor neo-antigens.

While mapping specific CDR3 sequences is beyond the current limitations of the platform, we reasoned that variable (V)-gene usage from V(D)J-recombination events would vary between samples in a manner reflecting differences in lymphocyte clonotype composition. To validate this approach, we compared patient-matched and time-matched samples between the Xenium data and their corresponding scRNA-seq counterparts. Tumor samples from the same patient taken at the same clinical timepoint—pre- or post-PD-1 blockade—should show overlapping patterns of V-gene usage in the scRNA sequencing vs. Xenium in situ analyses. Indeed, this was true for both TCR alpha and TCR beta chains, where mismatched samples had no correlation in overlapping usage and matched samples demonstrated enrichment in shared V-gene expression patterns (Fig. 3*A*). Interestingly, for BCRs, only the IG heavy chain and not the IG light chain displayed this matched pattern (Fig. 3*B*). Therefore, we proceeded with using the IG heavy chain alone for BCR clonotype tracking.

To examine functionally relevant T and B cell interactions, we analyzed spatial transcriptomics data for coclustering of T and B cell clonotype pairs—as defined by the dominant V-gene usage—using neighborhood enrichment analysis, which calculates the degree of spatial cooccurrence between selected cell types (26). We then examined the sizes of T and B clonotypes in our samples as a function of spatial colocalization. As expected, T and B cell clones with no spatial colocalization lacked any correlation in clonotype sizes (Fig. 3*C*). Interestingly, specifically in aPD-1-treated specimens, we observed significant correlation between B cell clone size and the number of paired, spatially colocalized T cell clones. This suggests that PD-1 blockade may promote the expansion of interacting T and B cells.

Previous studies have highlighted the importance of TLSs in successful responses to PD-1 inhibitors (4–6, 10–12). The presence of TLSs could potentially confound our analyses of BCR diversity within the tumor microenvironment. To address this issue, we evaluated hematoxylin and eosin staining of our specimens, and while we observed tumor-infiltrating lymphocytes, there were no clearly formed TLSs evident in any of our samples (*SI Appendix*, Fig. S4). This suggests that BCR clonotype diversity represents an important phenomenon independent of histologically distinguishable ectopic lymphoid structures. Given the absence of TLSs within the tumors, we hypothesized that regional draining lymph nodes may also be a critical site for the development of the T/B cell interactions that we observe in our data.

In two patients, tissue samples from both posttreatment tumors and the tumor-draining lymph nodes were available for analysis. We examined the presence of specific T and B cell clonotype pairs in tumors and regional lymph nodes. We find that T/B cell clonotype pairs that were colocalized in both tumor and lymph nodes were approximately 2 to 10 times more likely to be in both locations compared to noncolocalized clonotypes, which tended to be found exclusively in either tumor or lymph node (Fig. 3 *D*–*F*). This overlap was more pronounced in patient su001 who experienced excellent long-term response and remission of disease compared to patient su005 whose disease was progressing at last available follow-up.

We then examined the phenotypes of these “shared” clonotypes enriched in both tumor and lymph node, which appeared to be trafficking between these sites. In lymph node samples, T cells from these shared clonotypes exhibited higher expression of the proliferation marker *MKI67* compared to other cells (Fig. 3*G*). *CD69* expression, an indicator of recent activation, was observed in shared T cell clonotypes in both patients; however, it was only significantly elevated in the tumor specimen of su001, who achieved remission. Finally, *ENTPD1* (encoding CD39, a marker associated with tumor-reactive T cells) was elevated only in the tumor specimens of su001. These findings highlight the nuanced interpatient differences in PD-1 inhibitor responses and suggest a model by which B cell clones support the expansion and activation of specific T cell clones in lymph nodes, leading to more effective tumor clearance (Fig. 3*H*). The interpatient variation in how nodal clonal interactions turn into a successful tumor-reactive T cell response deserves further investigation.

While direct cytotoxic activity against tumor cells is driven by CD8+ T cells, various modes of support for these antigen-specific CD8+ T cells have been demonstrated. Recent studies have identified an immune cell triad of CD4+ T cells, CD8+ T cells, and dendritic cells that coalesce to reprogram CD8+ T cells for tumor clearance (27). Given that B cells, like dendritic cells, function as professional antigen-presenting cells, we hypothesized that the relationship we observe between B cells and CD8+ T cells may also be influenced, in part, by a supportive, colocalized CD4+ T cell interaction. Our coembedded spatial transcriptomics dataset, incorporating aPD-1-exposed tissue samples, uniquely enables the study of this phenomenon.

To quantitatively assess the presence of cell cluster triads, we defined a metric we call “*Triad Occurrence*” (*Materials and Methods* and *SI Appendix*, Fig. S7*A*) that measures the cooccurrence probability of having a third cell type in proximity to an existing proximal cluster pair. As a proof-of-concept, we first tested this measurement on our malignant cell clusters since these clusters of tumor cells exist in well-structured tumor islands and may represent phylogenetically related daughter cells. Compared to a triad of three cell types with no expected colocalization, these malignant cell clusters showed a significant propensity to colocalize, validating the utility of this analysis to detect the occurrence of cell cluster triads (*SI Appendix*, Fig. S7 *B* and *C*).

Next, we tested CD4+ T cell/Activated CD8+ T cell/Dendritic cell triads, which showed increased intratumoral coclustering in agreement with recent studies [*SI Appendix*, Fig. S7*D*, (27)]. In our prior study, we noted parallel development of follicular helper T cells (Tfh) and CD8+ T cells. Given our observations of B cell involvement in T cell activation in regional lymph nodes, we asked whether there might be a B cell/Tfh/Activated CD8+ T cell triad present in our patient samples. Indeed, we observe cooccurrence of this B cell/Tfh/Activated CD8+ T cell triad, but interestingly, specifically in regional lymph node samples, suggesting that a substantial portion of this interaction happens in the tumor-draining nodes (*SI Appendix*, Fig. S7 *E* and *F*).

### Trajectory of Induced BCR Diversity Correlates with Postimmunotherapy Survival in Multiple Cancer Types.

**Fig. 4.**
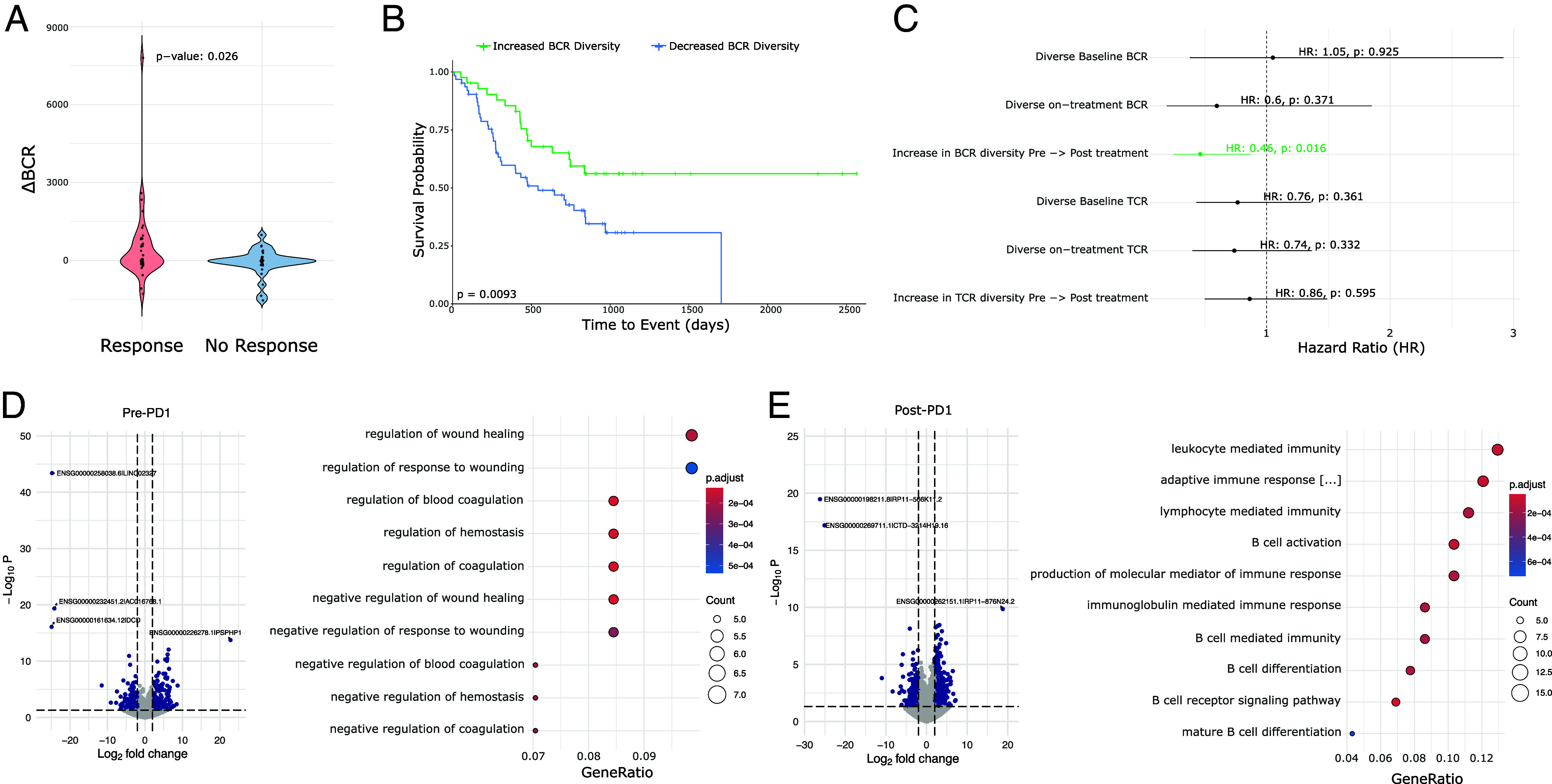
Meta-analysis of BCC, glioblastoma, melanoma, and HNSCC reveals a general prognostic role for aPD1-induced BCR diversity. (*A*) ∆BCR clonotype count from pre-PD1 inhibitor to post-PD-1-inhibitor of patients with progressed tumors vs. nonprogressed tumors. (*B*) Kaplan–Meier curve of overall survival for aggregate cancer patients stratified by induced BCR expansion from PD-1 inhibitor. (*C*) Hazard ratios of overall survival for aggregate cancer patients by BCR or TCR clonotype. An increased clonotype diversity is defined by a greater number of clonotypes detected in post-PD-1 treated tumors compared to prior. Diversity in the case of static measurements at baseline or on-treatment is defined as an above-median BCR or TCR clonotype count. (*D*, *Left*) Volcano plot of differentially expressed genes in pretreatment tumors that undergo induced BCR expansion vs. those without. (*Right*) GO-terms enriched in pretreatment tumors that undergo induced BCR expansion vs. those without. (*E*, *Left*) Volcano plot of differentially expressed genes in posttreatment tumors that undergo induced BCR expansion vs. those without. (*Right*) GO-terms enriched in posttreatment tumors that undergo induced BCR expansion vs. those without.

To determine whether the correlation between BCR diversity and survival is BCC-specific or represents a more generalizable phenomenon following checkpoint blockade, we aggregated data from published studies that include pre- and postimmunotherapy tumor sequencing and clinical outcomes. Identified studies meeting our criteria included head and neck SCC, melanoma, and glioblastoma, which we combined with our existing BCC dataset (7, 28–30). We extracted BCR clonotypes from these studies and categorized the patients based on the induction of BCR diversity following checkpoint blockade using the same criteria established in our earlier analysis. Across this aggregated dataset which included cancers of various embryonic origins, induced BCR diversity was associated with better clinical response (Fig. 4*A*). In the cohort of glioblastoma patients where progression-free survival data were available, higher BCR diversity was associated with prolonged progression-free survival, specifically in patients treated with PD-1 inhibitor in the neoadjuvant setting, whereas their treatment-naive counterparts with a scheduled, adjuvant course showed no significant difference (*SI Appendix*, Fig. S8). In the combined cohort of 93 patients (8 BCC, 12 Glioblastoma, 12 HNSCC, 61 Melanoma) across these four cancer types, overall survival was significantly better in patients exhibiting induced BCR clonal diversity following checkpoint blockade (Fig. 4*B*) (HR = 0.46 95% CI 0.245882 to 0.8678724, log-rank *P* = 0.0093).

Prior studies have suggested the potential prognostic importance of static BCR clonotype measurements (11, 16, 31), however, others have failed to detect significant associations (32). We therefore sought to evaluate this in our aggregated dataset. Consistent with previous reports, we observed a trend suggesting protective effects of higher static BCR diversity, particularly when measured in treatment-exposed tumors (Fig. 4*C*); however, in our multicancer cohort, the dynamic trajectory of induced BCR clonotypes following checkpoint blockade was more directly related to survival outcomes and was the only criterion that reached statistical significance.

### A Machine-Learning Model Predicts the Trajectory of BCR Clonotype Diversity from Pretreatment Tumor Gene Expression.

Although induced BCR diversity is a potentially important prognostic factor for immunotherapy response, measuring it in routine practice is challenging. Calculating induced BCR diversity requires adequate tumor specimens for sequencing from both the pretreatment tumor as well as from posttreatment tumors. While obtaining multiple sequential samples is practical in certain circumstances—such as when tumors are easily accessible (e.g., cutaneous neoplasms), or when neoadjuvant checkpoint blockade precedes a planned surgical resection attempt—in most cases, sequential sampling would expose the patient to additional, unplanned procedural risks. Therefore, it would be beneficial to understand whether this information could be derived solely from the baseline data from pretreatment tumors, thus providing more timely data for clinical decision-making while simultaneously avoiding the risks of additional tissue sampling.

To address this, we reannotated our multicancer cohort based on the patients’ eventual change in BCR diversity. Interestingly, pretreatment tumors that respond to PD-1 therapy with an induced increase in BCR diversity were enriched for genes related to the coagulation cascade (Fig. 4*D*). While studies have linked coagulation with the function of PD-1 inhibitors, to our knowledge this is not a known positive prognostic signature (33). High endothelial venules—specialized postcapillary structures known for their presence in secondary lymphoid organs—are known to enhance lymphocyte recruitment (34). These data pose a possibility that there may be specific vascular structures or signatures that prime the tumor for improved lymphocyte turnover, but this requires further, separate investigation. As expected, the posttreatment tumors with increased BCR diversity were enriched for gene expression relating to B cell function including “B cell activation,” “BCR signaling pathway,” and “lymphocyte/leukocyte mediated immunity” (Fig. 4*E*).

We hypothesized that these differentially expressed genes may be diagnostically important in identifying which tumors are primed for induced BCR diversity. To leverage this possibility, we developed a machine-learning model using this gene expression profile as input to categorically classify potential BCR responses to checkpoint blockade. We trained this neural network model on the 117 identified differentially expressed genes, achieving a 92.3% accuracy on a 20% withheld set of pretreatment tumor specimens (*Materials and Methods* and Dataset S3). This result suggests that baseline tumor gene expression may contain sufficient information to predict induced BCR expansion.

There are however limitations to this approach that need to be addressed. First, since the data in the training and validation sets originated from the same cohort, the validation does not account for real-world variability introduced by varying sample collection protocols and standards. Second, while induced BCR diversity is mechanistically interesting, it is not clinically actionable absent a direct link to treatment outcomes. To address both limitations, we subjected the model to a more challenging two-step inference problem: predicting clinical outcomes using only baseline tumor RNA sequencing data from a completely independent dataset. The model was completely naive to this new dataset, simulating the challenges of applying the model to a new cohort. Additionally, to ensure that the model was not simply identifying a nonspecific positive prognostic signature but rather one specific to checkpoint blockade, we included a control cohort of patients treated with nonimmunotherapy approaches including chemotherapy, radiation, and targeted molecular therapies who had no opportunity to induce a checkpoint inhibitor-specific mechanism of response. We identified cohorts of melanoma patients in published data and The Cancer Genome Atlas Program meeting these criteria and proceeded with modeling on these cohorts (35–37).

**Fig. 5.**
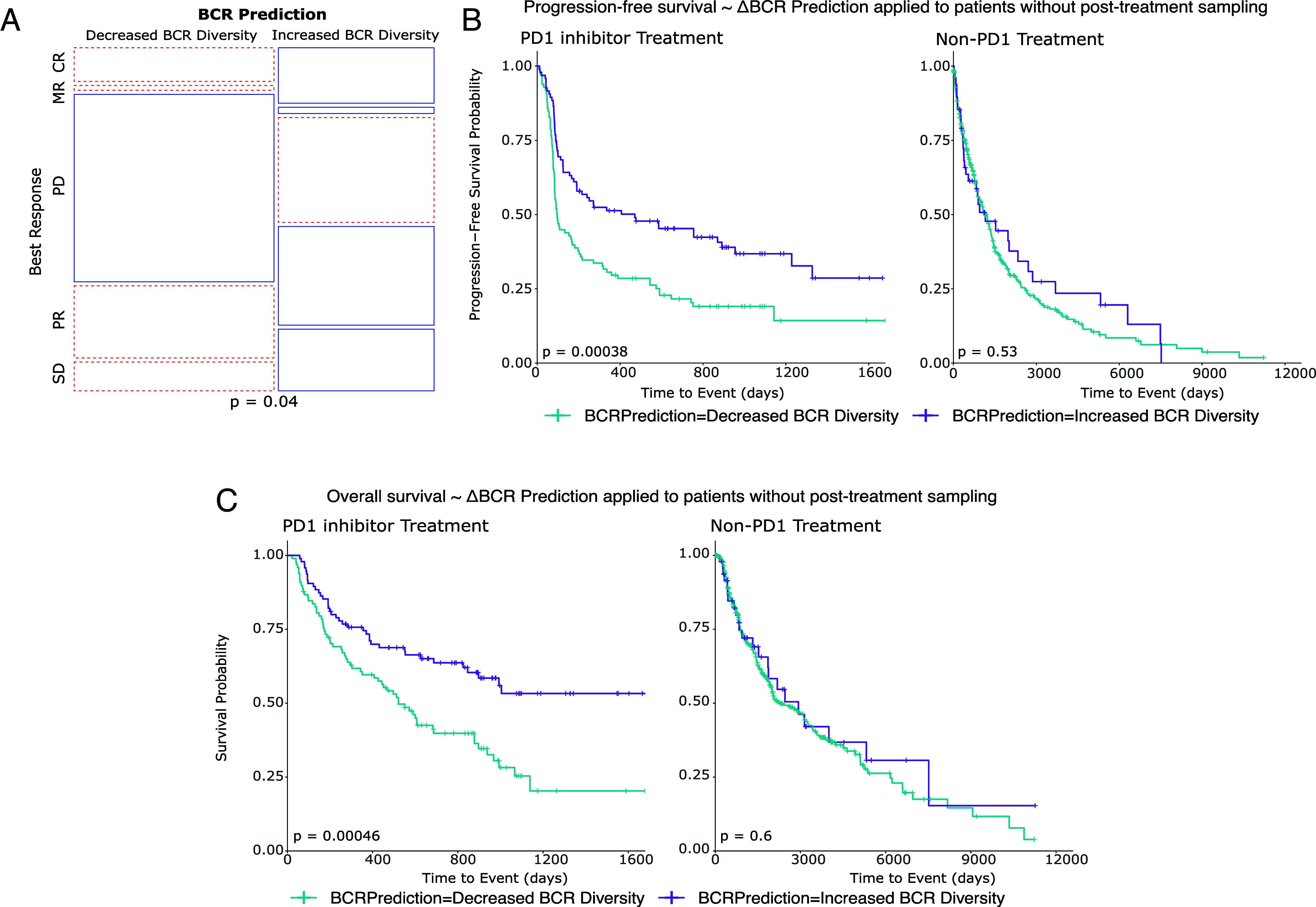
Pretreatment tumor RNA sequencing can be leveraged to model aPD-1-induced BCR diversity and clinical outcomes. (*A*) Prediction model applied to a new melanoma patient cohort. Mosaic plot of clinical response by RECIST stratified by model prediction of induced BCR clonotype expansion. BR: Best response, CR: Complete response, MR: Marginal response, PD: Progressed disease, PR: Partial response, SD: Stable disease. (*B*) Kaplan–Meier curve of progression-free survival stratified by model prediction of induced BCR clonotype expansion in melanoma patients treated with PD1 inhibitor (*Left*) and other, non-PD1 therapies (*Right*). (*C*) Kaplan–Meier curve of overall survival stratified by model prediction of induced BCR clonotype expansion in melanoma patients treated with PD-1 inhibitor (*Left*) and other, non-PD-1 therapies (*Right*).

Among the melanoma patients undergoing checkpoint blockade, those whose baseline tumors were predicted by the model to increase BCR diversity following treatment showed higher proportions of clinical responses (including marginal, partial, and complete responses), while those predicted not to have an increase in BCR diversity had higher proportions of progressive disease (Fig. 5*A*). Additionally, patients with predicted increases in BCR diversity had both superior progression-free survival (log-rank *P* = 0.00038) and overall survival (log-rank *P* = 0.00046) compared to their counterparts in whom the model predicted no increase in BCR diversity (Fig. 5 *B* and *C*). Supporting the checkpoint inhibitor-specific function of the model, patients who underwent treatment with non-PD-1 modalities did not show stratification by survival outcomes based on the model’s predictions (log-rank *P* = 0.53 and *P* = 0.60 for PFS and OS, respectively). These results support the possibility that the prognostic value of induced BCR clonotype diversity could be inferred without repeat on-treatment tissue sampling.

## Discussion

Immunotherapy has emerged as a leading treatment option for many advanced cancers. Its potential is evident in the positive clinical responses and cases of long-term remission that can be induced by checkpoint blockade therapies. The promise, however, is juxtaposed against many cases of inadequate response and an incomplete understanding of the mechanisms underlying treatment failure. The effectiveness of immunotherapy on a patient-by-patient basis arises from an ecosystem of interactions between the tumor, the patient’s immune state, their environment, among other factors (38).

In this study, we collected data under real-world circumstances to pair clinical response with underlying biology. We find that one potential outcome of checkpoint blockade therapy is the induction of BCR clonotype diversification. This diversification appears to support the activation and proliferation of CD8+ T cells in regional lymph nodes that then traffic to the tumor to enhance tumor clearance. The successful induction of BCR clonotype expansion is closely linked to patient outcomes, such as overall survival, not only in our cohort of BCC patients but additionally in cohorts of multiple cancer types. These findings support previously hypothesized mechanisms of B cell antitumor activity through their unique abilities to both clonally expand and present antigen (39) and agree with other studies highlighting the importance of tumor-infiltrating CD8+ T cells in improved cancer survival (40). Agents that promote B cell diversification, such as the CD40 or OX40 pathways, may directly impact this mechanism.

Despite these encouraging findings, our study has limitations including its retrospective nature and focus on specific malignancies. Future studies on prognostic use should be validated in a prospective manner, and studies with larger populations and more diverse tumor types would help clarify the generalizability of these mechanistic findings. Nevertheless, we hope this study advances the field in seeking better prognostic tools for cancer patients undergoing checkpoint blockade and guides improvements in immunotherapies for nonresponders to current regimens (39, 41).

## Materials and Methods

Human subject research in this study was approved by the Stanford University Administrative Panels on Human Subjects in Medical Research under IRB protocol number 18325. Written, informed consent was obtained from all participants, and participants were given the option to withdraw involvement at any time. All patients had histologically proven advanced or metastatic BCC not suitable for surgical resection at the time of study enrollment. Patients were treated with pembrolizumab 200 mg every 3 wk or cemiplimab 350 mg every 2 wk. Additional human subject methodology is presented in *SI Appendix*.

## Supplementary Material

Appendix 01 (PDF)

Dataset S01 (CSV)

Dataset S02 (CSV)

Dataset S03 (CSV)

## Data Availability

RNA sequencing analyzed in this study was obtained from the Gene Expression Omnibus (GEO), European Nucleotide Archive (ENA), and dbGAP at the following accessions: GSE123813 (7), GSE121810 (29), phs000452.v3.p1 [(35), only gene counts accessed], PRJEB23709 (36), GSE91061 (28), and GSE179730 (30). For the melanoma control dataset, patient sequencing and clinical data were downloaded from the cancer genome atlas Skin Cutaneous Melanoma PanCancer Atlas using cBioportal. Patients with PD-1 inhibitors in their treatments were excluded for the non-PD-1 melanoma control cohort. Xenium data associated with this publication are available at GSE291246.
