# Supplementary material for: Induced B cell receptor diversity predicts PD-1 blockade immunotherapy response
Source: Proc Natl Acad Sci U S A. 2025 May 2;122(18):e2501269122. doi: 10.1073/pnas.2501269122 (PMC12067265; doi:10.1073/pnas.2501269122)
Supplement: Supplementary file 1 — Appendix 01 (PDF) [file pnas.2501269122.sapp.pdf]

PNAS

Supporting Information for
Induced B Cell Receptor Diversity Predicts PD-1 Blockade Immunotherapy Response

Yonglu Che MD PhD[1], Jinwoo Lee MD PhD[1], Farah Abou-Taleb BS[1], Kerri E. Rieger MD PhD[1,2], Ansuman T. Satpathy MD PhD[2] , Anne Lynn S. Chang MD[1], Howard Y. Chang MD PhD[1,2,3,4]

Affiliations:
1. Department of Dermatology, Stanford University School of Medicine, Redwood City, CA, USA
2. Department of Pathology, Stanford University School of Medicine, Stanford, CA, USA
3. Department of Genetics, Stanford University School of Medicine, Stanford, CA, USA
4. Howard Hughes Medical Institute, Stanford University School of Medicine, Stanford, CA, USA

Correspondence to: Anne Lynn S. Chang MD and Howard Y. Chang MD PhD
Email: alschang@stanford.edu and howchang@stanford.edu

This PDF file includes:

Supporting text
Figures S1 to S8
Legends for Datasets S1 to S3
SI References

Other supporting materials for this manuscript include the following:

Datasets S1 to S3

Materials and Methods

Human subjects

As previously described (1), data collected in this study was approved by the Stanford University Administrative Panel on Human Subjects in Medical Research (IRB protocol number 18325). Our institution complies requirements for protection of human subjects including 45 CFR 46. All participants provided written informed consent. The patients included in this follow-up had histologically-confirmed advanced or metastatic basal cell carcinoma not suitable for surgical resection at the time of enrollment. Patients underwent treatment with pembrolizumab (200mg every 3 weeks) or cemiplimab (350mg every 2 weeks). Exclusion criteria included prior exposure to checkpoint blockade agents, systemic immunosuppression, exposure to radiotherapy, and use of any other anti-cancer agents within 4 weeks of first biopsy for scRNA-seq. Patient su003 had a distant >50 year prior history of radiation for acne at an unrelated site, patient su004 had adjuvant radiation for a squamous cell carcinoma at a unrelated site two years prior to enrollment, and patient su006 received unspecified radiation for a childhood medulloblastoma > 50 years prior to enrollment. The remaining patient did not undergo radiotherapy prior to enrollment. The medical records for these patients were accessed in November 2023 to supplement long-term survival data not available at the time of prior data publication. Where there was a clearly documented date of death, this was used as the endpoint for survival analyses. In the absence of a date of death, the last confirmed medical contact in our medical record was used as the date of censorship. Two separate investigators were responsible for updating this clinical record with the second investigator blinded to any molecular data analyses at the time of medical record review to reduce bias.

Tumor collection, library preparation, and sequencing

The in-depth protocol for the collection and processing of human patient samples is as previously described (1). In brief, tissue samples were collected during clinical visits at Stanford Hospital and Clinics. Written consent was obtained prior to each tissue collection. The local area of tissue sampled was marked, photographed, and anesthetized with lidocaine. The subsequent tissue processing was performed as previously described for each downstream application. Biopsy specimens were obtained using a 4mm punch biopsy device. Wound care was performed as clinically indicated for the collection sites.

For single-cell RNA sequencing, the samples were processed using the 10x Single Cell Immune Profiling Solution Kit according to the manufacturer's instructions. The resulting libraries were sequenced on either an Illumina NextSeq or HiSeq 4000 to a minimum sequencing depth of 25,000 reads per cell with the following read lengths: Read1 – 26, i7 index: 8, Read 2: 98. The sequencing reads were aligned to the GRCh38 reference genome and quantified using Cellranger count (10x Genomics, Version 2.1.0). For clinical care specimens, biopsy sites were prepared and anesthetized in the same manner. Specimens were collected through a tangential biopsy technique with a curved sterile blade (Derma Blade Shave Biopsy Instrument), punch biopsy tool, or scalpel depending on the context of specimen collection. Tissues were immediately submerged in formalin 10% and then subsequently embedded in paraffin. Tissue sections were cut as required for clinical diagnosis and the remaining blocks were preserved in long-term storage. These tissue blocks were requested at the time of this study for additional analysis as described below.

Single cell RNA sequencing analysis

ScRNA-seq data from (1) were obtained from the Gene Expression Omnibus and the supplementary data of the publication. These data were re-analyzed using Seurat (Version 5.0.3). Identical to prior cutoffs, cells with less than 200 genes detected or greater than 10% mitochondrial RNA content were excluded. Cell cluster annotations were performed based on the expression of known marker genes as previously reported, summarized below.

Cell Type	Marker Genes
T cells	CD3G, CD3D, CD3E, CD2
CD8+ T cells	CD8A, GZMA
$CD4^+$ T cells and T_{reg} cells	CD4, FOXP3
NK cells	KLRC1, KLRC3
B cells	CD19, CD79A
Plasma cells	SLAMF7, IGKC
Macrophages	FCGR2A, CSF1R

Dendritic cells	FLT3
Plasmacytoid dendritic cells	CLEC4C
Fibroblasts	COL1A2
Myofibroblasts	MCAM, MYLK
Cancer-associated fibroblasts	FAP, PDPN
Malignant cells	EPCAM, TP63
Endothelial cells	PECAM1, VWF
Melanocytes	PMEL, MLANA

ScRNA-seq data was analyzed as previously described (1). Cell clustering was performed with Seurat using the same cell lineage markers previously described. The cluster identities were largely in agreement to prior, with the updated analysis showing differences only in subclustering of malignant cell clusters and the identification of a small plasma blast population. Receptor-ligand and signaling pathway relationships between single cells were analyzed using Cellchat (Version 2.1.0) (2). Diffusion maps and pseudotime analyses were created with the R package Destiny (Version 3.16.0) (3).

TCR and BCR clonotypes

T-cell receptor and B-cell receptor repertoires were identified using TRUST4 (4) (Version 1.0.13). TRUST4 was run on the scRNA-seq FASTQ files with default parameters to extract V(D)J sequences from unassembled reads. These clonotypes were defined based on V/J gene usage and CDR3 sequences. A unique clonotype was considered only if there were three or more supporting reads. Analysis of this dataset required the use of Stanford's "Sherlock" high performance computing cluster. Further downstream analyses were performed using the R programming language (R Development Core Team, http://www.r-project.org/) and using the R package Immunarch (https://immunarch.com/, Version 0.9.1).

Correlation analysis of BCR diversity-associated gene expression

To identify genes whose expression levels are associated with BCR clonotype counts, we performed a correlation analysis using the scRNA-seq dataset. To additionally understand how individual cell clusters were contributing to this gene expression correlation and whether there was a dominant cell cluster which might be influenced by tissue BCR diversity, we implemented a cluster elimination strategy as a form of regression analysis, where cell clusters were systematically excluded and the correlations recalculated to assess each cluster's impact on gene-BCR correlations.

Gene expression counts were obtained from the log-normalized counts in the Seurat data. BCR clonotype counts derived from TRUST4 analysis of the tissue specimens were annotated in the metadata of the Seurat object. We computed Pearson correlation coefficients between the expression of each gene and the BCR clonotype counts present in the tissue specimen.

The cluster elimination strategy was implemented by creating a new Seurat object that systematically excluded each cell cluster from the dataset and re-performed the correlation calculations described above. We defined the change/delta in correlation as the difference between the Pearson correlation of the data with the specific cell cluster of interest excluded vs. that of all the cell clusters without exclusion. Visualization of this data was performed using ComplexHeatmap (Version 2.18.0).

Cell cluster triad analysis

Cell clusters in the spatial transcriptomics dataset were annotated using the nearest-neighbor approach in the ENVI latent space described separately. The CD4+ T cell population was subdivided into a CXCR5+ population based on detectable probe counts that we refer to as Tfh cells. Specifically for this analysis we propose a new metric that quantified the frequency of a third co-localizing cell type in proximity to a co-localizing cluster pair of interest. We term this metric the Triad Occurrence as defined below. The distance threshold d is set to be the approximate diameter of an average cell. The p-value was calculated by generating an empiric null distribution over 10,000 iterations and comparing the true observed value to this null distribution. All-by-all configurations were avoided to minimize confounding from multiple hypothesis testing.

$$\text{Triad Occurrence} = \frac{\sum_{i=1}^{n} I\left(dist\left(A(i),B(i)\right) \le d \wedge dist\left(A(i),C(i)\right) \le d\right)}{n}$$

$n: Total\ occurences\ of\ cluster\ A\ (origin\ cell)$
$d: distance\ threshold. In\ this\ case\ 10\mu m$
$dist(A(i), B(i))$: $distance\ between\ the\ i-th\ occurence\ of\ cell\ belonging\ to\ cluster\ A\ and\ the\ nearest\ cell\ belonging\ to\ cluster\ B$
$dist(A(i), C(i))$: $distance\ between\ the\ i-th\ occurence\ of\ cell\ belonging\ to\ cluster\ A\ and\ the\ nearest\ cell\ belonging\ to\ cluster\ C$
$I(x)$: $Indicator\ function. I(x) = 1\ if\ x\ is\ true\ and\ I(x) = 0\ if\ x\ is\ false$

Statistical analysis
Time to event analyses (PFS, OS, BCC/SCC risk) were performed using the Kaplan-Meier method. Hazard ratios were calculated using the Cox proportional hazards model. Contingency tables were analyzed using the chi-square test. P-values are as reported in the main text with the default reporting being the p-value from the log-rank test unless otherwise stated.

Realignment of RNA sequencing differential gene expression analysis
FASTQ sequences obtained from GEO were realigned to the GRCh38 reference genome using HISAT2 (Version 2.1.0) (5) using default parameters. Gene quantification was performed using Stringtie (Version 2.2.1) (6). Differential gene expression was conducted using DEseq (Version 1.12.3) (7). Genes with an adjusted p-value < 0.05 and an absolute log2 fold change > 1 were considered significantly differentially expressed. Where FASTQ files were not available, the aligned counts from the referenced datasets were used directly.

Machine Learning of baseline tumor characteristics for BCR predictions
To Predict BCR clonotype expansion based on baseline tumor gene expression, we developed a neural network model using the Keras (Version 2.13.0) library in R. Linear regression-based models and random forest models were unable to generate predictions that exceeded random chance. The input features were the 117 differentially expressed genes identified from pre-treatment tumors where the comparison cohorts were those tumors that went on to have BCR diversity increase vs. those without BCR diversity increase. Modeling with similar architecture using whole genome transcript counts was unsuccessful, possibly due to overfitting.

The dataset was split into 80% training and 20% validation sets using stratified random sampling to maintain proportional representation. The neural network architecture was designed to minimize complexity and therefore avoid overfitting. The final architecture consisted of:

- An input layer with 117 units corresponding to the input features.
- A hidden layer with 64 units and ReLU activation.
- A dropout layer with a rate of 0.5.
- A second hidden layer with 32 units and ReLU activation.
- A dropout layer with a rate of 0.5.
- An output layer with 1 unit and linear activation for regression output.

The model was compiled using the Adam optimizer and used a mean squared error loss function (then categorized on above or below mean prediction value). Training was performed over 800 epochs and early stopping implemented based on validation loss. Model performance was evaluated using the root mean squared error on the categorical assignments of BCR clonotype change and the coefficient of determination on the validation dataset.

Xenium In Situ panel design
Genes were selected for Xenium In-Situ based on multiple criteria. First, we automatically included all genes from our prior study that were required for cell type or cell state identification. Next, we sought the assistance of the 10x design team to assist with the design of custom probes specific to individual V(D)J genes. These probes were validated internally by 10x Genomics. We next identified the most differentially expressed genes present in our prior scRNA-seq dataset and included the maximum number of these genes in order of degree of differential expression up to the maximum allowed capacity of 480 genes at the time of design. The final design was assessed using the custom design panel from 10x genomics. Two panels – "Human Skin (aging)" and Human Skin (normal/inflamed)" were used as design references in the quality control. 475 out of the 480 selected genes were within the recommended expected expression range to avoid optical crowding, however, given our assessment of the expression in scRNA-seq data, all 480 were retained for the final panel design.

Xenium In Situ

Xenium In Situ Gene Expression assays were run according to the manufacture's (10x Genomics) instructions. Custom probe kits and accompanying reagents were ordered from 10x Genomics and stored at their recommended temperature until use, in our case within 6 months.

A 3 mm diameter core was punched from the region of interest (ROI) of each FFPE donor block and placed into its designated position within the recipient block. Cores were arranged in a 2x4 or 2x5 sample array to align with the available Xenium slide area. The recipient block, containing the specimens, was placed into a paraffin embedding station to fill any gaps and secure the cores. Five-micron sections of the TMA block were then cut using a microtome and mounted onto slides. Quality control (QC) was performed by staining and examining test sections to confirm that the cores were intact and properly oriented. Care was taken to ensure that the microarray grid did not interfere with the slide fiducials during Xenium slide preparation. Microarrays were sectioned directly onto the Xenium slide in five-micron sections immediately prior to slide processing. Following sectioning, the Xenium slides were air-dried under a fan for 30 minutes and then baked at 42°C for 3 hours. The slides were subsequently stored overnight in a desiccated container at room temperature.

Deparaffinization of the tissue slides was performed as follows: 1) Incubation at 60C for 2h. 2) Cooling tissue to RT for ~7min. 3) Immersion in xylene x 10 min x 2. 4) Immersion in 100% ethanol x 3 min x2. 5) Immersion in 96% ethanol for 3 min x2. 6) Immersion in 70% ethanol x 3 min. 7) Immersion in nuclease-free water for 20s. 8) Wash with 1x PBS.

Decrosslinking of the tissue slides was performed as follows: 1) Incubation of Decrosslinking buffer (see manufacturer's instructions) at 80C x 30min then 22C for 10 min. 2) Wash with 1x PBS-T x 1 min x 3.

Custom probes were resuspended in TE buffer according to manufacturer instructions immediately prior to use. Probe hybridization was performed overnight at 50C. Post-hybridization washes were performed with PBS-T at RT then again at 37C x 30miin. The probe ligation reaction was performed at 37C for 2h followed by PBS-T washes at RT. Amplification was performed at 30C for 2h and followed by TE buffer washes at RT. Autoflorescence quenching was performed to chemically mitigate background florescence according to the manufacturer instructions. Prepared slides were loaded onto the Xenium Analyzer instrument running Xenium Onboard Analysis v2.0.

To improve the chances of capturing accurate cell boundaries, all Xenium slides were additionally run with multi-modal cell segmentation. This kit contains a variety of cell stains including DAPI for nuclear staining/segmentation, ATP1A1, E-Cadherin, and CD45 for establishing membrane boundaries, 18S ribosomal RNA labels to detect the cytoplasm, and alphaSMA/Vimentin for interior protein staining. The default isotropic nuclear expansion distance is set as 5 µm. Standard H&E staining was performed immediately after the decoding and imaging. Briefly, the post-run slide was immersed in Quencher Removal Solution (10x Genomics) and then washed with Mili-Q water. Staining was performed with Hematoxylin, Bluing solution, and then Eosin according to manufacturer instructions.

Critical data files of the output were exported off the Xenium Analyzer Instrument. Image registration and initial data exploration was performed in Xenium Explorer before being exported to other community-based, open-source methods as separately described.

Kits and reagents from 10x Genomics were used for the preparation of Xenium slides and the Xenium Analyzer run, including: Xenium Slides & Sample Prep Reagents (PN-1000460), Xenium Decoding Consumables (PN-1000487), Xenium Decoding Reagents (PN-1000461), and Xenium Cell Segmentation Staining Reagents (PN-1000661).

Neighborhood enrichment analysis

Neighborhood enrichment was run for the Xenium data using Squidpy (Version 1.6.0) (8). Cells expressing V-genes were categorized by the highest-expressing V-gene within their segmented cell boundary. These assignments were then used as surrogate cell clusters using the sq.gr.spatial_neighbors function within Squidpy. The neighborhood enrichment analysis calculates an enrichment z-score based on the proximity on the

connectivity graph of these clonotype assignments. A z-score of 2 was used as a cutoff for spatially significant enrichment for downstream analyses.

BCC cohort survival analysis

Patients su001 to su008 from Yost et al., 2019 were included in the analysis. Patient su009 to su013 were excluded due to the absence of BCR data (T-cell isolates only were obtained). Patient su004 who lacked detectable immunoglobulin clones in both pre-and post-PD-1 treatment samples was classified as a BCR non-expanded patient. To minimize noise contributed by non-specific BCR calls and to focus the analysis on clones more likely to be functionally relevant in the tumor, a minimum threshold of 3 counts per clones was established to define a significant BCR clone. This represented the same threshold cutoff used in our prior study.

Patient were categorized into two groups based on the dynamic change from pre- to post-PD-1 treated tumors.

BCR Expanded: Patients exhibiting an increase in BCR clone counts in post-PD-1 treatment tumors compared to pre-PD-1 treatment tumors.
BCR non-Expanded: Patient exhibiting no change or decrease in BCR clone counts in post-PD-1 treatment tumors compared to pre-PD-1 treatment tumors.

The change in BCR clone counts was defined as follows:

$\Delta BCR = BCRpost\text{-}treatment - BCRpre\text{-}treatment$

Given that there are multiple metrics used to assess BCR clonotype diversity, we tested the robustness of our methodology in measuring ΔBCR by using multiple established metrics of clonotype diversity including chao1, div, gini, and d50. We find that the aggregate agreement of these metrics result in the same BCR Expanded/non-Expanded categorization as the primary methodology used above.

Survival times were computed from the date of first PD-1 inhibitor exposure until either the date of death – when available – or the last follow-up date for censored observations. Kaplan-Meier curves were generated using a combination of the R packages survival (Version 3.5.8), Survminer (Version 0.4.9), and plotted with either ggplot2 (Version 3.5.0) or using Graphpad Prism 10. Survival distributions were compared between groups using the log-rank test. Hazard ratios and 95% confidence intervals were estimated using the Cox proportional hazards regression model to assess the impact of BCR clonal expansion on overall survival.

ENVI cointegration of scRNA-seq and Xenium

ENVI (Version 0.3.6) (9) was run using the CPU-based computation paramenters given the large memory requirements of our data-set. Dependencies for this analysis were installed according to the instructions recommended in the package documentation. Prior to analysis both scRNA-seq and Xenium data were re-normalized to a target sum of 10,000 counts per cell and log-transformed. To ensure a consistent latent space, ENVI was run with all spatial samples represented in each model initialization. For each subset, the model was trained using the default parameters. Due to memory constraints, the data was batched into 5 random subsets and processed individually with consistent results obtained from each subset.

Dimensionality reduction was performed using Uniform Manifold Approximation and Projection (UMAP) with parameters set to 200 nearest neighbors and a minimum distance of 0.05. Cell type predictions for the spatial transcriptomics dataset were created by identifying the nearest-neighbors with respect to Euclidean distance in the high-dimensional latent space generated by ENVI of known cell cluster assignments in cells originating from the scRNA-seq dataset (10, 11).

SpatialData analysis and Visualization

Xenium data were managed using a combination of Xenium Explorer and the python package SpatialData (Version 0.2.0). Xenium Explorer was used for registration of the RNA expression coordinates with the concurrent H&E staining of the same tissue. Given the arrayed format of the tissues, once the coordinates of the individual samples were determined, the data/images for each tissue sample were cropped to the correct areas and stored in Zarr format.

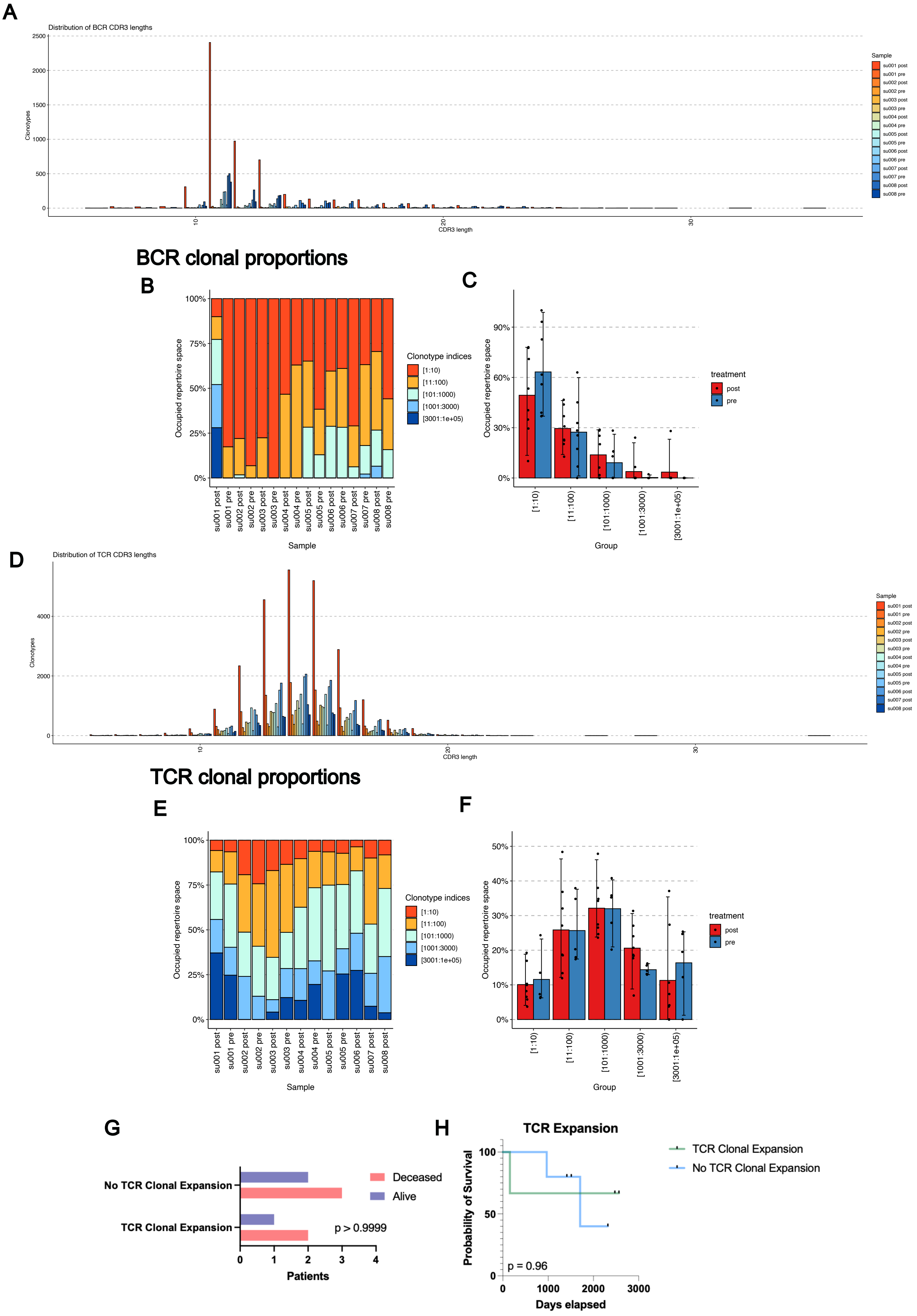

A
Distribution of BCR CDR3 lengths
Clonotypes
CDR3 length
Sample
su001 post
su001 pre
su002 post
su002 pre
su003 post
su003 pre
su004 post
su004 pre
su005 post
su005 pre
su006 post
su006 pre
su007 post
su007 pre
su008 post
su008 pre
BCR clonal proportions
B
C
Occupied repertoire space
Clonotype indices
[1:10)
[11:100)
[101:1000)
[1001:3000)
[3001:1e+05)
Sample
treatment
post
pre
Group
D
Distribution of TCR CDR3 lengths
TCR clonal proportions
E
F
G
No TCR Clonal Expansion
TCR Clonal Expansion
Deceased
Alive
p > 0.9999
Patients
H
TCR Expansion
Probability of Survival
Days elapsed
p = 0.96
TCR Clonal Expansion
No TCR Clonal Expansion

Supplementary Figure 1: BCR and TCR Clonotype Characteristics

A) Distribution of BCR CDR3 lengths by sample.
B) Clonal proportions of BCR clonotypes present in BCC patient specimens expressed as a percent of total.
C) Occupied repertoire space of BCR clonotypes present in BCC patient specimens stratified by sample pre vs. post PD1 inhibitor.
D) Distribution of TCR CDR3 lengths by sample
E) Clonal proportions of TCR clonotypes present in BCC patient specimens expressed as a percent of total.
F) Occupied repertoire space of TCR clonotypes present in BCC patient specimens stratified by sample pre vs. post PD1 inhibitor.
G) Contingency table of patient status at last clinical follow up vs. the presence of significant clonal expansion in any T cell subset previously described in Yost et al. 2019.
H) Kaplan-Meier curve of overall survival for our BCC patient cohort stratified by presence or absence of T cell subset expansion.

A

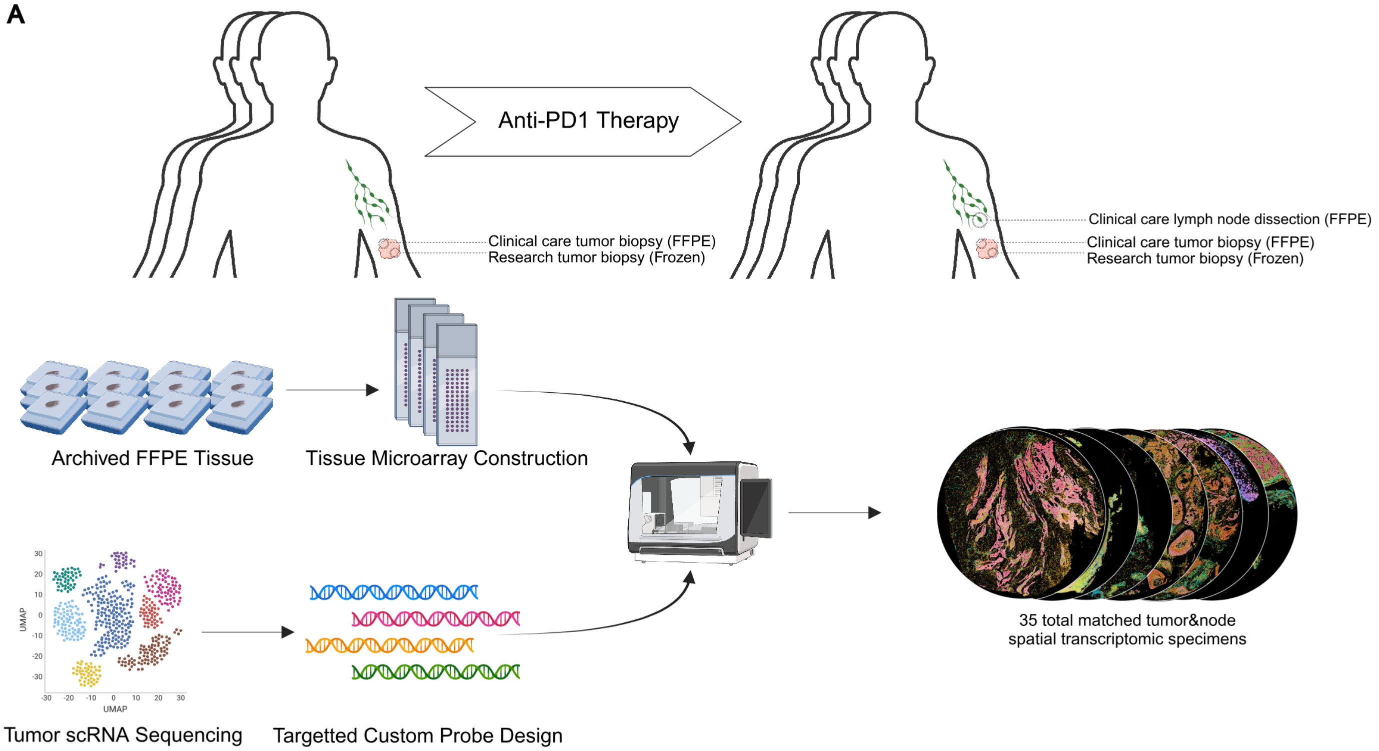
Anti-PD1 Therapy
Clinical care tumor biopsy (FFPE)
Research tumor biopsy (Frozen)
Clinical care lymph node dissection (FFPE)
Clinical care tumor biopsy (FFPE)
Research tumor biopsy (Frozen)
Archived FFPE Tissue
Tissue Microarray Construction
35 total matched tumor&node
spatial transcriptomic specimens
UMAP
UMAP
Tumor scRNA Sequencing
Targetted Custom Probe Design

B

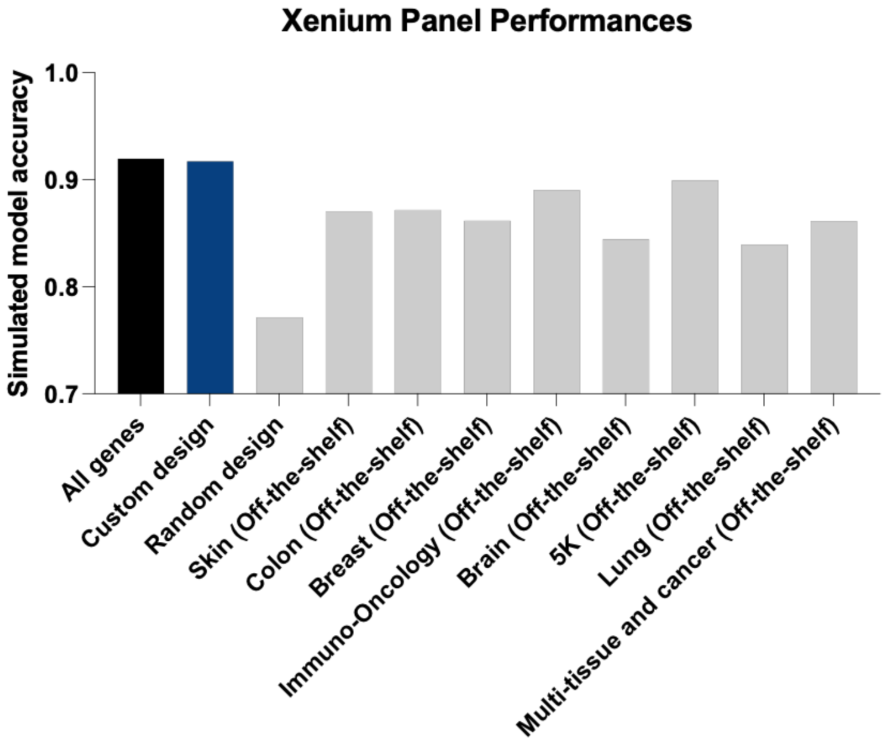
Xenium Panel Performances
Simulated model accuracy
1.0
0.9
0.8
0.7
All genes
Custom design
Random design
Skin (Off-the-shelf)
Colon (Off-the-shelf)
Breast (Off-the-shelf)
Immuno-Oncology (Off-the-shelf)
Brain (Off-the-shelf)
5K (Off-the-shelf)
Lung (Off-the-shelf)
Multi-tissue and cancer (Off-the-shelf)

Supplementary Figure 2: Xenium In-Situ Experimental Design and Probe Design Validation

A) Schematic diagram of Xenium in situ experimental design using archived specimens.
B) Model performance of a neural net classifier trained on cell type prediction from scRNA-seq data. A theoretical maximum of model performance was established by allowing full gene expression data to be used in training (black) while all other models were restricted to the genes available in the annotated Xenium panels The custom design (blue) is the 480 gene manually curated gene panel used for our analysis, the random design is a panel of 480 randomly selected genes while all other panels are pre-designed panels offered by 10x genomics.

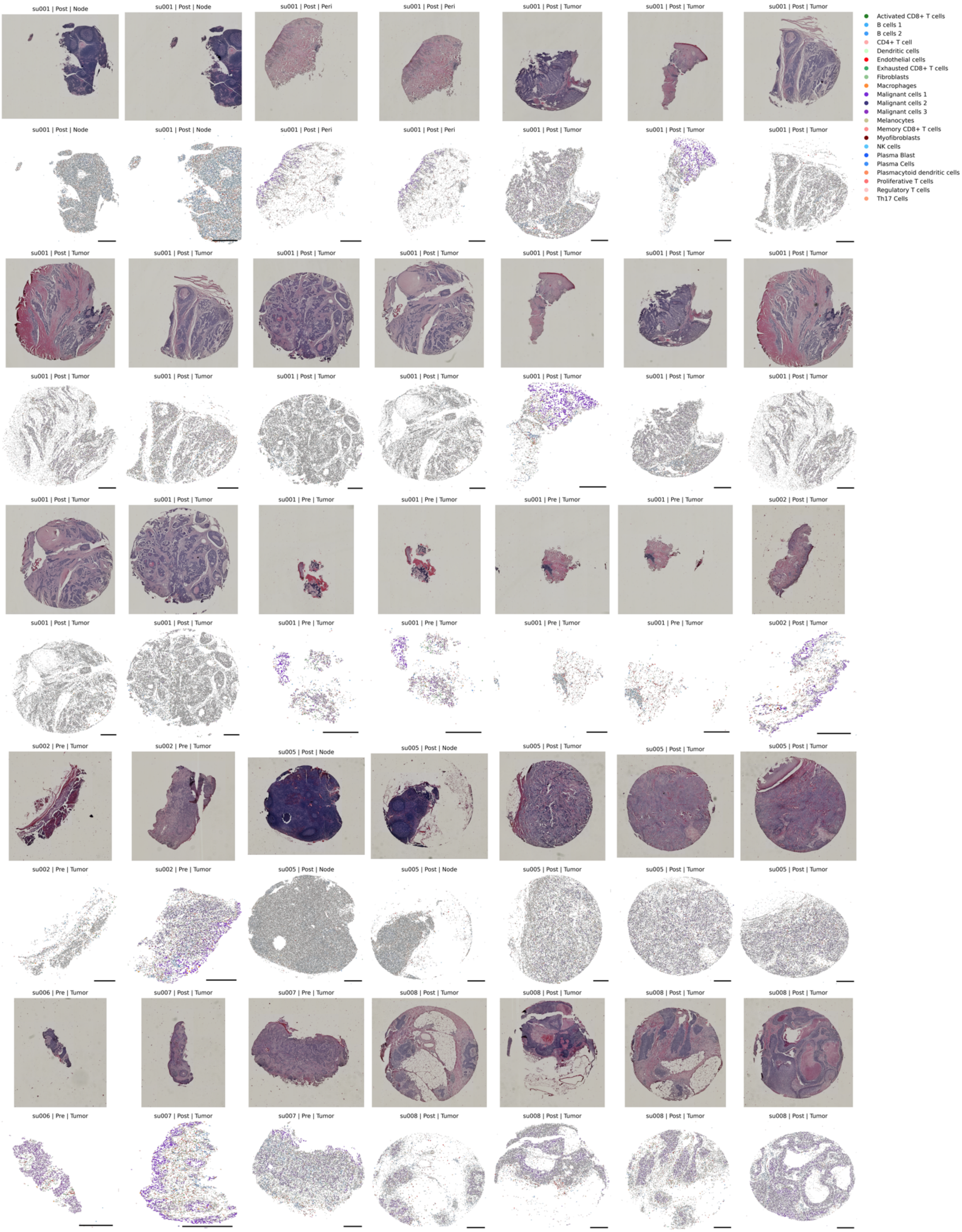

su001 | Post | Node
su001 | Post | Node
su001 | Post | Peri
su001 | Post | Peri
su001 | Post | Tumor
su001 | Post | Tumor
su001 | Post | Tumor
su001 | Post | Node
su001 | Post | Node
su001 | Post | Peri
su001 | Post | Peri
su001 | Post | Tumor
su001 | Post | Tumor
su001 | Post | Tumor
su001 | Post | Tumor
su001 | Post | Tumor
su001 | Post | Tumor
su001 | Post | Tumor
su001 | Post | Tumor
su001 | Post | Tumor
su001 | Post | Tumor
su001 | Post | Tumor
su001 | Post | Tumor
su001 | Post | Tumor
su001 | Post | Tumor
su001 | Post | Tumor
su001 | Post | Tumor
su001 | Post | Tumor
su001 | Post | Tumor
su001 | Post | Tumor
su001 | Pre | Tumor
su001 | Pre | Tumor
su001 | Pre | Tumor
su001 | Pre | Tumor
su002 | Post | Tumor
su001 | Post | Tumor
su001 | Post | Tumor
su001 | Pre | Tumor
su001 | Pre | Tumor
su001 | Pre | Tumor
su001 | Pre | Tumor
su002 | Post | Tumor
su002 | Pre | Tumor
su002 | Pre | Tumor
su005 | Post | Node
su005 | Post | Node
su005 | Post | Tumor
su005 | Post | Tumor
su005 | Post | Tumor
su002 | Pre | Tumor
su002 | Pre | Tumor
su005 | Post | Node
su005 | Post | Node
su005 | Post | Tumor
su005 | Post | Tumor
su005 | Post | Tumor
su006 | Pre | Tumor
su007 | Post | Tumor
su007 | Pre | Tumor
su008 | Post | Tumor
su008 | Post | Tumor
su008 | Post | Tumor
su008 | Post | Tumor
su006 | Pre | Tumor
su007 | Post | Tumor
su007 | Pre | Tumor
su008 | Post | Tumor
su008 | Post | Tumor
su008 | Post | Tumor
su008 | Post | Tumor
Activated CD8+ T cells
B cells 1
B cells 2
CD4+ T cell
Dendritic cells
Endothelial cells
Exhausted CD8+ T cells
Fibroblasts
Macrophages
Malignant cells 1
Malignant cells 2
Malignant cells 3
Melanocytes
Memory CD8+ T cells
Myofibroblasts
NK cells
Plasma Blast
Plasma Cells
Plasmacytoid dendritic cells
Proliferative T cells
Regulatory T cells
Th17 Cells

Supplementary Figure 3: H&E and Xenium In-situ of Primary Patient Specimens

Patient tissue specimens sent for Xenium in situ analysis. Top panels represent H&E staining of the identical tissue section run for Xenium. Bottom panels are spatial cell boundaries (grey) colored by cell type predictions generated through the previously described nearest neighbor approach in the ENVI latent space. Scale bars are 0.5mm. Xenium cells are downsampled to 40% for visualization.

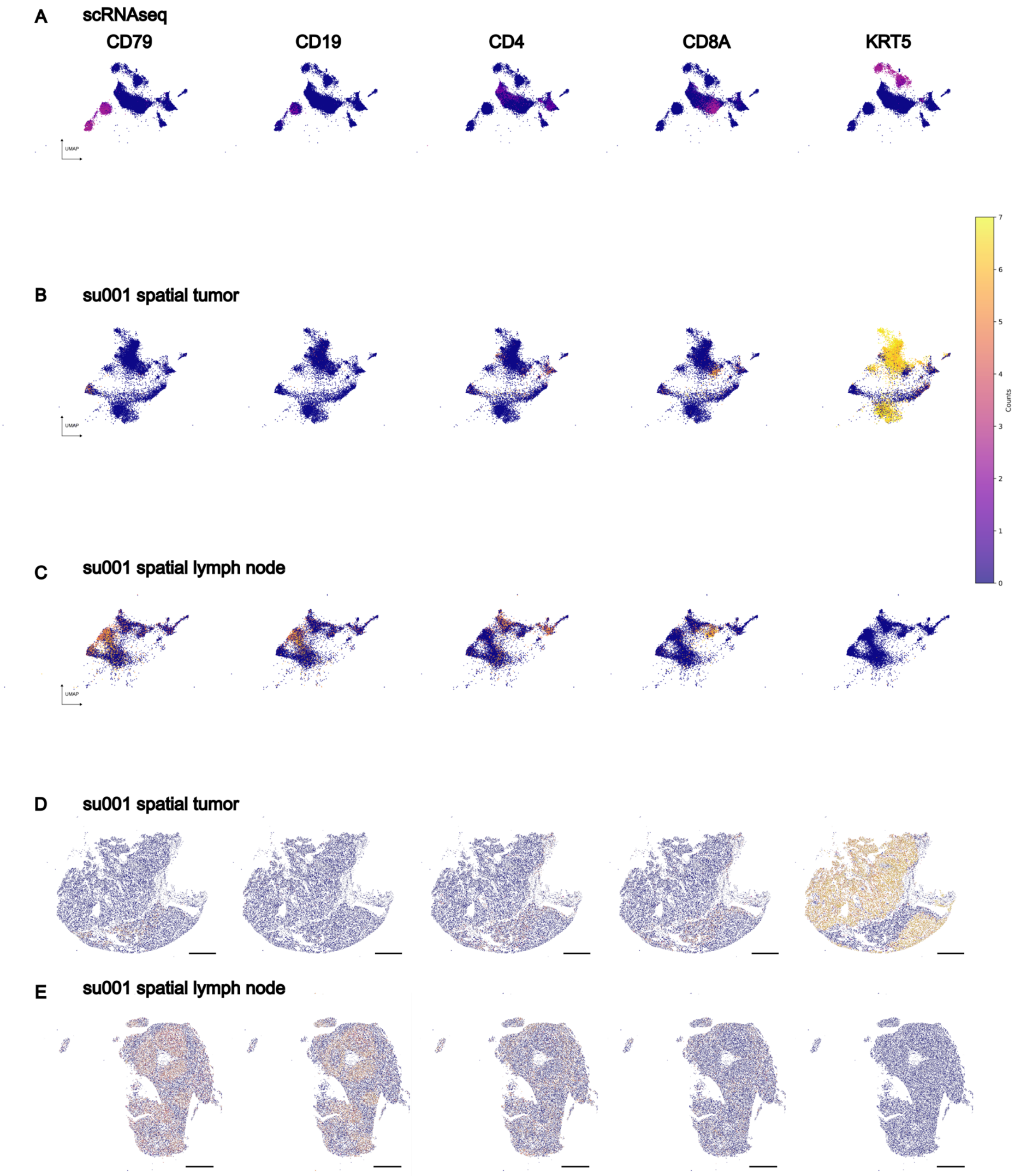
A
scRNAseq
CD79
CD19
CD4
CD8A
KRT5
UMAP
B
su001 spatial tumor
C
su001 spatial lymph node
D
su001 spatial tumor
E
su001 spatial lymph node
Counts
0
1
2
3
4
5
6
7

Supplementary Figure 4: ENVI Latent Space and Spatial Representation of Example Tumor and Lymph Node

A) UMAPs of scRNA-seq cells B) Xenium tumor sample cells and C) Xenium draining lymph node cells colored by expression of select cell type markers in the shared ENVI latent embeddings.
D) Spatial plots of the sample shown in B.
E) Spatial plots of the sample shown in C. Scale bars are 0.5mm.

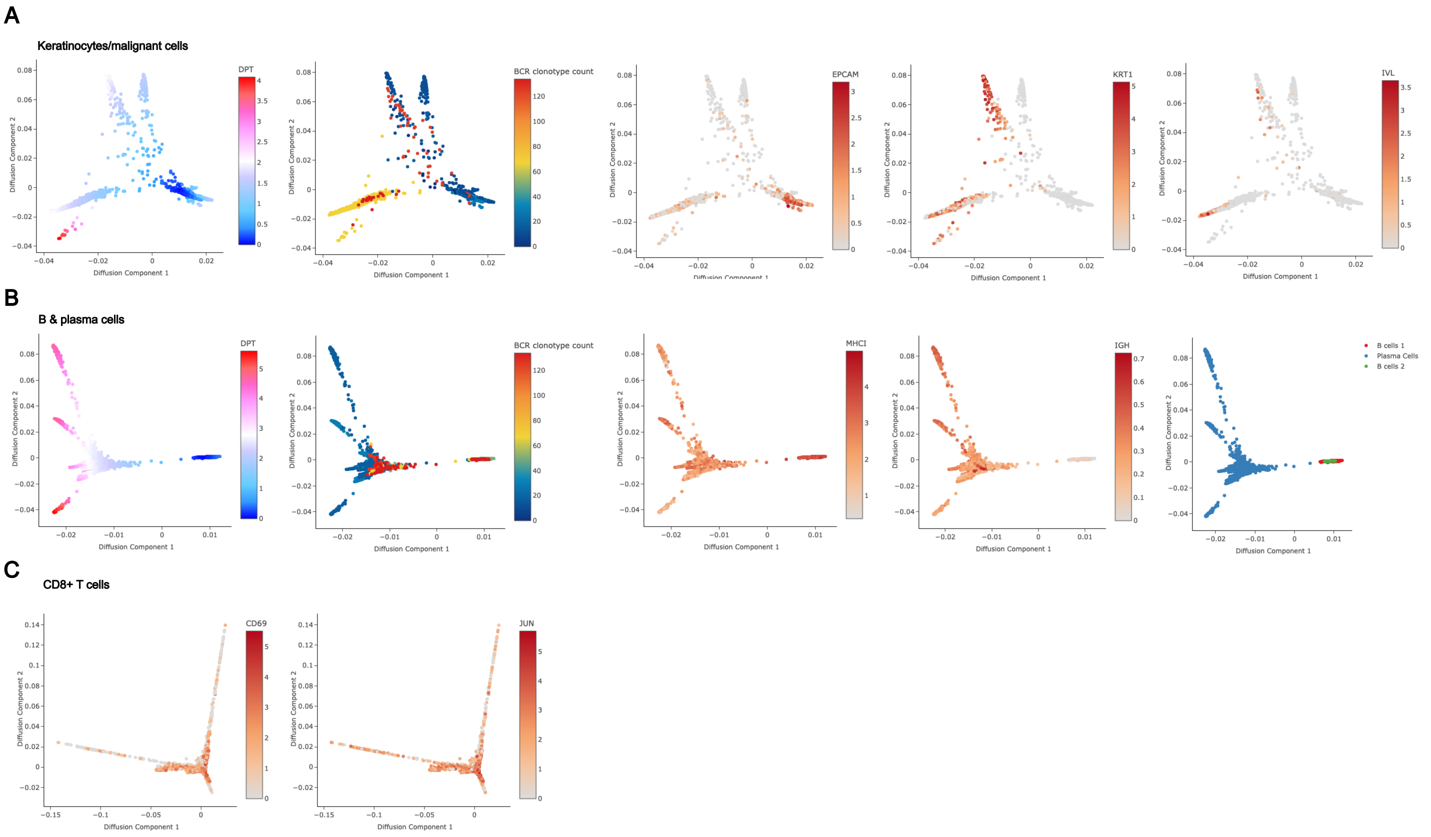

Supplementary Figure 5: Diffusion Plots of Tumor and Immune Cell Clusters Reveals Increased Activated CD8+ T-cell Representation and Decreased Tumor Burden in BCR-rich Samples

A) Keratinocyte cells (identified in this study broadly as malignant cells) diffusion plot. DPT stands for diffusion pseudotime. The BCR clonotype count for each cell plotted is defined as the number of BCR clonotypes present in the tissue sample from which the plotted cell originated, representing the BCR diversity of its environment. Expression of EPCAM (BerEP4) a marker of basal cell carcinomas and progenitor keratinocytes, KRT1 an early differentiation marker, and IVL a late differentiation marker plotted per cell.

B) B and plasma cells aggregated diffusion plot. MHC-I expression is defined as the mean expression of all class I MHC genes per cell. IGH expression is defined as the mean expression of all heavy chain Ig genes per cell.

C) CD8+ T cells diffusion plot. Expression of CD69 and JUN, markers of TCR-induced activation plotted per cell.

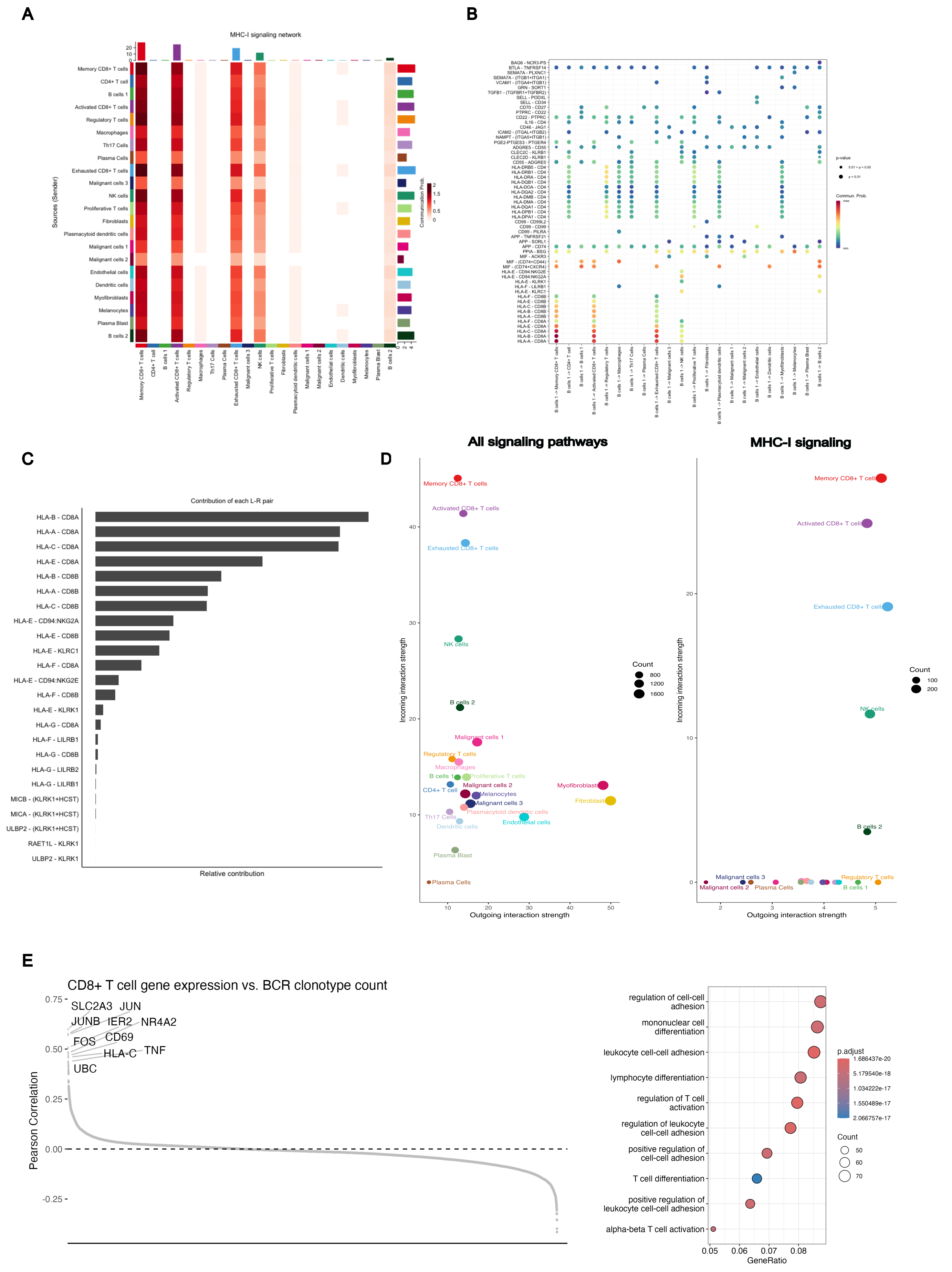

A
MHC-I signaling network
Sources (Sender)
Communication Prob.
Memory CD8+ T cells
CD4+ T cell
B cells 1
Activated CD8+ T cells
Regulatory T cells
Macrophages
Th17 Cells
Plasma Cells
Exhausted CD8+ T cells
Malignant cells 3
NK cells
Proliferative T cells
Fibroblasts
Plasmacytoid dendritic cells
Malignant cells 1
Malignant cells 2
Endothelial cells
Dendritic cells
Myofibroblasts
Melanocytes
Plasma Blast
B cells 2
B
p-value
Commun. Prob.
C
Contribution of each L-R pair
HLA-B - CD8A
HLA-A - CD8A
HLA-C - CD8A
HLA-E - CD8A
HLA-B - CD8B
HLA-A - CD8B
HLA-C - CD8B
HLA-E - CD94:NKG2A
HLA-E - CD8B
HLA-E - KLRC1
HLA-F - CD8A
HLA-E - CD94:NKG2E
HLA-F - CD8B
HLA-E - KLRK1
HLA-G - CD8A
HLA-F - LILRB1
HLA-G - CD8B
HLA-G - LILRB2
HLA-G - LILRB1
MICB - (KLRK1+HCST)
MICA - (KLRK1+HCST)
ULBP2 - (KLRK1+HCST)
RAET1L - KLRK1
ULBP2 - KLRK1
Relative contribution
D
All signaling pathways
MHC-I signaling
Incoming interaction strength
Outgoing interaction strength
Count
800
1200
1600
100
200
E
CD8+ T cell gene expression vs. BCR clonotype count
Pearson Correlation
SLC2A3
JUN
JUNB
IER2
NR4A2
FOS
CD69
HLA-C
TNF
UBC
regulation of cell-cell adhesion
mononuclear cell differentiation
leukocyte cell-cell adhesion
lymphocyte differentiation
regulation of T cell activation
regulation of leukocyte cell-cell adhesion
positive regulation of cell-cell adhesion
T cell differentiation
positive regulation of leukocyte cell-cell adhesion
alpha-beta T cell activation
GeneRatio
p.adjust
1.686437e-20
5.179540e-18
1.034222e-17
1.550489e-17
2.066757e-17
Count
50
60
70

Supplementary Figure 6: CellChat Analysis of B-Cell/T-Cell Signaling

A) Heatmap of communication probabilities in MHC-I signaling pathways between cell clusters.
B) Significant communication pathways originating from cluster B cell 1 representative of B outgoing B cell signaling.
C) Relative contribution of each ligand-receptor pair to MHC-I signaling strength between B cell clusters and Activated CD8+ T cells.
D) Outgoing and incoming signaling strength per cell cluster for all signaling pathways (left) and MHC-I signaling pathways (right) calculated using CellchatDB.
E) Pearson correlation between gene expression in CD8+ T cells vs. the BCR clonotype count in the respective tissue sample. Each point is the correlation of a single gene, and the plot it ordered from most positive correlation to most negative correlation.
F) GO-Terms enriched in genes significantly correlated with BCR clonotype count in CD8+ T cells (defined as adjusted p-value <0.05).

Supplementary Fig. 7: Immune Triads Involving B cells and Activated T cells

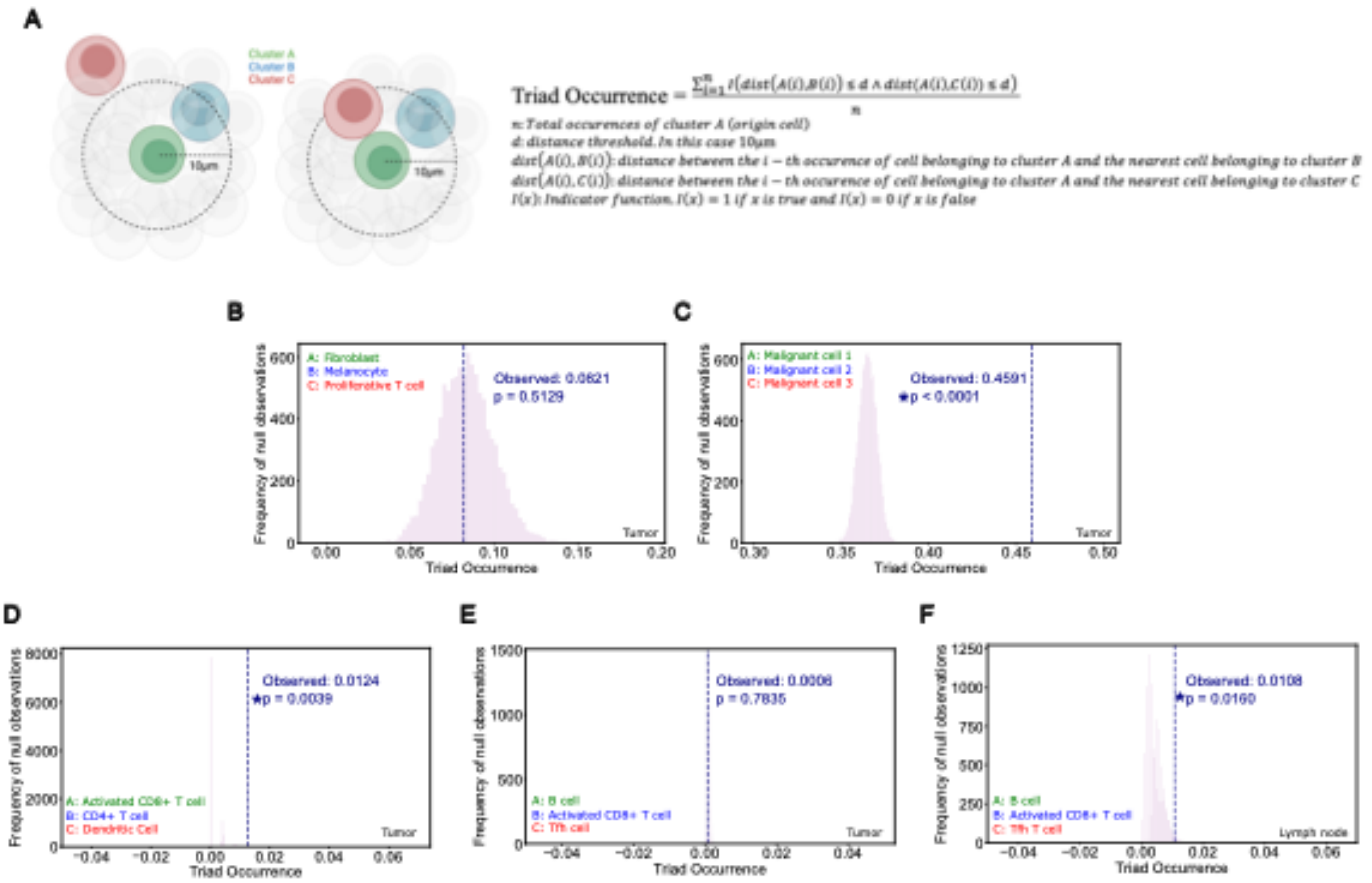

Supplementary Figure 7: Immune Triads Involving B cells and Activated T cells

A) Schematic and equation of spatial cell triad calculation. The Triad Occurrence is defined as the proportion of existing co-localized pairs of two cell types (Cluster A and B) that also have a proximal third cell type interaction (Cluster C).
B) Fibroblast/Melanocyte/Proliferative T cell Triad Occurrence; negative control in aPD1-treated tumors.
C) Malignant cell 1/Malignant cell 2/Malignant cell 3 Triad Occurrence; positive control in aPD1-treated tumors.
D) Activated CD8+ T cell/CD4+ T cell/Dendritic cell Triad Occurrence in aPD1-treated tumors.
E) B cell/Activated CD8+ T cell/Tfh Triad Occurrence in aPD1-treated tumors.
F) B cell/Activated CD8+ T cell/Tfh Triad Occurrence in aPD1-treated draining lymph nodes.

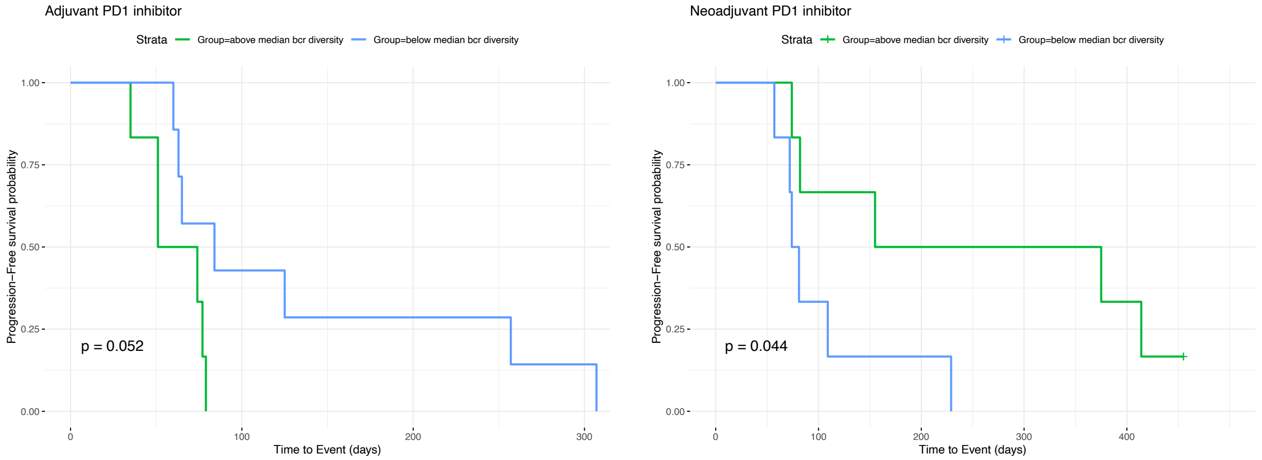

Supplementary Figure 8: Progression-free Survival In Glioblastoma as a Function of PD-1 Inhibitor-induced BCR Diversity

Left: Progression-free survival in glioblastoma patients who underwent resection then started on adjuvant PD1 inhibitor. Tumor collection on which BCR clonotype analysis was performed was prior to PD1 exposure (Cloughesy et al. 2019). P values reported for log rank test.

Right: Progression-free survival in glioblastoma patients exposed to neoadjuvant PD1 inhibitor followed by surgical resection.

Dataset S1 (separate file). Clinical Outcomes

Dataset S2 (separate file). Xenium In-Situ Panel

Dataset S3 (separate file). BCR diversity DEgenes

SI Appendix References

1. K. E. Yost, *et al.*, Clonal replacement of tumor-specific T cells following PD-1 blockade. *Nat Med* **25**, 1251–1259 (2019).

2. S. Jin, *et al.*, Inference and analysis of cell-cell communication using CellChat. *Nat Commun* **12**, 1088 (2021).

3. L. Haghverdi, F. Buettner, F. J. Theis, Diffusion maps for high-dimensional single-cell analysis of differentiation data. *Bioinformatics* **31**, 2989–2998 (2015).

4. L. Song, *et al.*, TRUST4: immune repertoire reconstruction from bulk and single-cell RNA-seq data. *Nat Methods* **18**, 627–630 (2021).

5. D. Kim, J. M. Paggi, C. Park, C. Bennett, S. L. Salzberg, Graph-based genome alignment and genotyping with HISAT2 and HISAT-genotype. *Nat Biotechnol* **37**, 907–915 (2019).

6. M. Pertea, *et al.*, StringTie enables improved reconstruction of a transcriptome from RNA-seq reads. *Nat Biotechnol* **33**, 290–295 (2015).

7. M. I. Love, W. Huber, S. Anders, Moderated estimation of fold change and dispersion for RNA-seq data with DESeq2. *Genome Biol* **15**, 550 (2014).

8. G. Palla, *et al.*, Squidpy: a scalable framework for spatial omics analysis. *Nat Methods* **19**, 171–178 (2022).

9. D. Haviv, *et al.*, The covariance environment defines cellular niches for spatial inference. *Nat Biotechnol* **43**, 269–280 (2025).

10. A. Janesick, *et al.*, High resolution mapping of the tumor microenvironment using integrated single-cell, spatial and in situ analysis. *Nat Commun* **14**, 8353 (2023).

11. Y. Hao, *et al.*, Integrated analysis of multimodal single-cell data. *Cell* **184**, 3573-3587.e29 (2021).
